# Effects of Geometric Sound on Brainwave Activity Patterns, Autonomic Nervous System Markers, Emotional Response, and Faraday Wave Pattern Morphology

**DOI:** 10.1155/2024/9844809

**Published:** 2024-03-29

**Authors:** Rona Geffen, Christoph Braun

**Affiliations:** ^1^Independent Scholar, Athens, Greece; ^2^Tübingen University, MEG-Center, Tübingen 72074, Germany; ^3^HIH Hertie Institute for Clinical Brain Research, Tübingen, Germany; ^4^CIMeC Center for Mind/Brain Sciences, University of Trento, Trento, Italy

## Abstract

This study introduces Geometric Sound as a subfield of spatial sound featuring audio stimuli which are sonic holograms of mathematically defined 3D shapes. The effects of Geometric Sound on human physiology were investigated through EEG, heart rate, blood pressure, and a combination of questionnaires monitoring 50 healthy participants in two separate experiments. The impact of Geometric Sound on Faraday wave pattern morphology was further studied. The shapes examined, pyramid, cube, and sphere, exhibited varying significant effects on autonomic nervous system markers, brainwave power amplitude, topology, and connectivity patterns, in comparison to both the control (traditional stereo), and recorded baseline where no sound was presented. Brain activity in the Alpha band exhibited the most significant results, additional noteworthy results were observed across analysis paradigms in all frequency bands. Geometric Sound was found to significantly reduce heart rate and blood pressure and enhance relaxation and general well-being. Changes in EEG, heart rate, and blood pressure were primarily shape-dependent, and to a lesser extent sex-dependent. Pyramid Geometric Sound yielded the most significant results in most analysis paradigms. Faraday Waves patterns morphology analysis indicated that identical frequencies result in patterns that correlate with the excitation Geometric Sound shape. We suggest that Geometric Sound shows promise as a noninvasive therapeutic approach for physical and psychological conditions, stress-related disorders, depression, anxiety, and neurotrauma. Further research is warranted to elucidate underlying mechanisms and expand its applications.

## 1. Introduction

Geometric Sound (GS) integrates sound projection with mathematical principles and geometric constants. Our hypothesis suggests that projections of geometric holograms of spatial sound exert specific effects on biological systems including human physiology. The resultant effect is attributed to the intrinsic mathematical information encoded within the projected GS shapes. This is theoretically achieved through a geometric feedback loop conveyed by sound wave vibrations. Any deviation from a nonmathematical ratio alignment among the projection components could compromise the integrity of the projected sonic hologram, which is crucial for the principle of a coherent geometric feedback loop.

In order to validate this hypothesis on objective measures, a series of preliminary exploratory experiments was conducted in the years 2017-2018, monitoring brainwave activity using electroencephalogram (EEG), heart rate (HR), blood pressure, behavioral response, and Faraday waves pattern morphology. A preliminary experiment in 2017 (EX1) monitored 20 healthy young adults for their response to GS. Multiple physiological markers including EEG, HR, blood pressure, and behavioral response were recorded. Results of the Autonomic Nervous System (ANS) were consistent with behavioral reports of enhanced relaxation and focus. In addition, interesting indications were shown to occur in the Alpha and Beta2 range following exposure to GS compared to standard Stereo sound (control) and Baseline (no sound). Faraday waves' pattern morphology analysis using the CymaScope instrument indicated a similar distinct response to GS. Following these results, a second experiment was conducted in 2018 (EX2) monitoring a larger participant group of 30 young adults and including additional GS shape. The primary objective of this experiment was twofold: to verify the EEG findings and to conduct a comprehensive examination of topographical changes in power and connectivity patterns. This experiment focused on EEG recordings and behavioral response and did not monitor biomarkers of the ANS. It is important to acknowledge that the initial study (EX1) was preliminary and, therefore, indicative. The confirmation of results in the second experiment (EX2) adds robustness, prompting us to present both studies together for a more comprehensive understanding.

The study originated from an artistic-scientific project titled “The Sound Is The Scenery (TSITS)” which investigated the connection between sound, geometry and mathematics with healing and well-being by immersing people in geometric structures of spatial sound and light [1]. This study launches the field of Geometric Sound. As a nascent field, we provide relevant data to acquaint the reader with its nature and interdisciplinary components. Following is a background introduction to the fields informing it.

Geometric structures and numeric sequences are widely spread in nature as functional frameworks for efficient energy distribution. Examples include Fibonacci sequence at the basis of nucleotide frequencies in human genome [2], geometric basis of chemical elements atomic structure [3], relation between prime numbers, and atomic structure in crystal-like materials [4], toroidal coupling of atomic information to mediate biological physical and emotional processes [5], and crystallization processes [6]. Mathematical sequences such as the Fibonacci sequence are found in growth algorithms in nature and in harmonic ratios and are maintained in general natural phenomena [7], for example, various flora constants [8], limbic and facial symmetries, and constructions [9]. Researchers assume that growth and alignment patterns in this sequence adheres strength and efficiency [8, 10]. The “Golden Ratio,” or Phi, which is easily derived from Fibonacci and Fibonacci-like sequences, is closely related to the construction of geometry [11] and was found to exist at the quantum level in magnetic resonance of atoms [12] and in Red Blood Cells (RBC) discoid structure [13]. Such mathematical constants allow similar geometric patterns to repeat on a range of scales in nature, for example pentagonal and hexagonal structures ranging from DNA molecule binding [14, 15] to planetary trajectories [16]. These examples show a distinct correlation of geometric symmetry with coherency and effectively form the foundation of the present study in which a geometric feedback loop is employed to impose and to entrain natural systems into coherency.

Sound is inherently related to geometry and mathematics with ordered symmetry of standing waves and their related mathematical ratios. Similar ratio patterns as those in the harmonic ratios of an octave were found in different natural phenomena such as the periodic table [17], planetary relations [18], colors [19], and brain waves [20]. An important example of how coherency is expressed in a geometric manner in nature is the propagation of sound waves, known as Chladni figures in its 2D expression [21], and Faraday Waves or Cymatics in its 3D expression [22–24]. Matter excited by sound frequencies can be visualized by the morphology of vibrational patterns in the form of symmetrical geometries. The patterns are known to be determined primarily by frequency, amplitude, and boundary condition of the observed medium [25]. Faraday waves were indicated to diagnose and differentiate cancer cells from healthy cells in brain tissues, healthy cells demonstrated to generate symmetrical patterns while cancerous cells were more prone to generate nonsymmetric, chaotic patterns [26]. Research suggests that water crystals are prone to form in symmetrical hexagonal structures when exposed to coherent stimuli, such as music, as opposed to a chaotic structure when exposed to incoherent or distorted stimuli such as noise [27, 28]. In the context of self-biofeedback to support self-regulation processes, it is interesting to note that sound researcher Hans Jenny was indicated to teach deaf children to improve their pronunciation by having them look at the cymatic patterns emerging from their own voice, hence seeing the dissonances in false pronunciation and the symmetry created by correct pronunciation [22]. Other documented positive effects of sound and music on various healing processes include improvement in physiologic and psychologic coping mechanisms [29] stress, depression and anxiety relief [30–32], advantageous effect on mood [33, 34], energy and physical state [35, 36], enhancement of RBC longevity [37], synchronization of brain waves [38–40], and enhancement of structural and cellular reaction of supporting hearing mechanisms in mice [41]. In accordance, medical sound and music interventions were found to positively improve various mechanisms such as hormone secretion [42], post-neurotrauma physiological and emotional symptoms [43–46], and support stress, and pain relief mechanisms [35, 47].

Nowadays, the use of music therapy in hospitals and schools is increasingly growing [48–52]. As our society becomes more accustomed to the use of music as a healing modality, the use of sound in this context is relatively new to western societies. However, sound medicine, also known as sound healing, is an ancient practice used throughout history in different cultures to relieve physical pain and emotional turmoil [53, 54]. Traditional sound medicine incorporates the use of sounds such as the human voice, nature sounds and various instruments including tuning forks, singing bowls, gongs and drums-all providing sound that is rich in frequencies and overtones. The natural mechanisms on which sound medicine relies upon are entrainment and resonance coherence of frequencies and rhythms. Notably, every sound is rhythmic as each frequency is derived from its vibrational oscillation. Although it is often assumed that sound is perceived only by our ears, its vibratory rhythmic oscillating pulsation, or subtle mechanical pressure, is perceived by our skin, bones, internal organs and cells [55]. Furthermore, natural living systems emit sound as part of their functional mechanism as well as depend on sound for healthy systemic maintenance. Commonly known examples include the sound of our breath, heart, and nervous system. Additional examples show that cocooned butterflies produce sound as part of their mechanical oscillation metamorphosis [56], plants are affected by sound as they grow [57, 58], sound of a healthy coral biosystem supports the rehabilitation processes of dead corals [59], cells emit sound and react with high pitched sound when agitated by toxic substances [60]. These examples demonstrate the core theme leading sound medicine of sequenced entrainment and revival of physiologic mechanical processes.

While growing in scope, there is still relatively very little scientific research on the physiological effects of sound medicine and most studies that explored its effects focused on behavioral evidence. For example, following sound meditation participants reported significantly less tension, anger, fatigue, and depressed mood, while feeling of spiritual well-being significantly increased [61]. Sound meditation was reported to result in significant effects of items such as physical relaxation, imagery, ineffability and positive mood across both live and recorded sessions [62]. High-resolution audio music box sound was reported to increase vigilance and relaxation, as recorded using EEG [63]. In addition there is accumulating evidence of the positive physiological effects of other relaxation practices such as mindfulness [64], meditation [65] and music therapy [30], as recorded by EEG [66–68] functional magnetic resonance imaging (fMRI) [69, 70] and Heart Rate Variability (HRV) [71, 72]. The well-documented positive effects of non-invasive relaxation practices on human health should be considered in accordance with the documented negative effects of stress and anxiety. This includes autoimmune disease and inflammatory processes [73–76], acute pain [77–79], various skin conditions [80], reproductive system failure [81–83], cardiovascular disease [84, 85], respiratory system failure [86–88], dysfunction in muscular dystrophy [89, 90], bone depletion [91], immune system deficiency [92], eruption of psychological conditions [76, 93, 94], and premature and enhanced aging symptoms [95–97].

Notably, while the use of alternative medicine such as sound medicine may be disregarded, there is solid scientific evidence confirming natural enhancements are as beneficial, and sometimes more beneficial than synthesized medications [98–101]. The means of employing music and sound medicine remain mostly traditional with relatively few prodigious technologies involved. A pioneer in the field of high-tech frequency-based sound medicine is Cyma Technologies [102]; other enterprises have employed tactile technology for neurotrauma rehabilitation [103]. While the use of high-resolution audio signals was shown to have an improved effect on human health [63], the use of high-end sound technologies is mostly sparse in this context. As such, advanced spatial sound technologies and audio systems which allow sound immersion are in general not recognised. Spatial sound is an emerging technology enabling precise sound localization and movement notation. It facilitates enhanced multidirectional sound experience and projection of audio holograms, to provide a 360-degree sound field. The effect of immersion provided by spatial sound systems exceeds the commonly known experience of listening, as it allows the entire body to be enclosed by directed sound vibrations. Notably, unlike the common perception of sound as a 2D waveform medium, sound is a spatial medium, spherical in nature and ever expanding [104]. Its spherical form contains a multiplicity of waves and their equivalent particle trajectories. New observations on Faraday waves patterns morphology following sound excitation conducted on the Cymascope instrument, suggest that sound is holographic by nature [105]. Importantly, observations on Faraday waves pattern morphology following sound excitation are in agreement with observations on pattern morphology following mechanical excitation [106–108], both demonstrating that patterns are crucially affected by the excitation frequency and boundary condition of the observed medium [25]. Accordingly, in many frequency-based physiological interventions, varied and distinct effects are reported following excitation by specific frequencies. A review paper by Bartel and Mosabbir [109] on possible mechanisms activated by sound on human health demonstrates discrete effects related to excitation by different sound and mechanical frequencies. A few examples include, 50 Hz sound stimulation and 1-2 Hz mechanic stimulation found to enhance blood flow [110, 111], 40 Hz sound stimulation found, among others, to impact cell differentiation [112–114] and improve motor impairments in Parkinson's Disease [115], and muscle treatment found to benefit from particular resonance frequencies [116].

This study suggests a non-invasive method of employing sound and mathematical constants to induce positive physical and emotional effects using spatial sound technology in combination with sound medicine.

## 2. Participants and Methods

**Figure 1:**
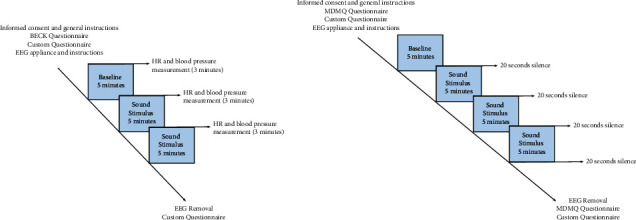
Experiments diagram plot. (a) EX1 diagram plot (2017). (b) EX2 diagram plot (2018).

Two separate experiments were conducted monitoring an overall of 50 participants. The first experiment in 2017 (EX1, Figure 1(a)) monitored 20 participants immersed in Sphere GS. For technical reasons 10 participants were additionally immersed in Pyramid GS. The second experiment in 2018 (EX2, Figure 1(b)) monitored 30 participants immersed in Pyramid, Cube and Sphere GS shapes. Across both experiments all participants were monitored for Baseline and Stereo (control) conditions. Participants were sitting in the center of the virtual shape projections at their base vertical level. Experiments were conducted within group comparisons and participants were exposed to all types of sound stimuli in a randomized order. EEG and ANS measurements were taken while participants were comfortably seated with eyes closed. The study was approved by the Ethics Committee of the Medical Faculty of Tübingen University. Experiments were conducted on 4DSOUND [117] at MONOM Center for Spatial Sound, Berlin.

### 2.1. Participants

A total of 50 participants were monitored in EX1 and EX2, of which 22 were female and 28 were male, with mean age being 26. In EX1, 20 participants were monitored (8 Female and 12 male, mean age 26). In EX2, 30 participants were monitored (14 female and 16 male, mean age 26). All participants were young adults (20–40) and signed an informed consent and waiver form prior to enrolling in the study. Participants declared not to suffer from any mental or health issues to their awareness and were not taking any medication regularly. Participants were instructed to sit with eyes closed for the entire experimental paradigm. Recruitment was conducted via an open call on social media and a random group of people was chosen from all applicants. The preferred research group for this preliminary study was healthy young adults in order to demonstrate possible effect on mundane situations as well as establish and validate baseline indication for safety of the method prior to possible studies on designated populations with more acute conditions.

### 2.2. Sound Stimuli

The study centered on one interval of an octave between the frequencies 272.2 Hz and 544.4 Hz played simultaneously and produced by unweighted tuning forks manufactured by Acutonics [118]. The selected frequencies are higher octaves of 136.1 Hz which is commonly used in sound meditation and sound medicine practices to induce a sense of grounding, relaxation and improved sense of wellbeing. Recent studies have confirmed the positive effect of 136.1 Hz on human emotional and physical biomarkers [119–121]. In order to facilitate technical needs and a coherent steadiness of the perceived audio signal by participants the use of 136.1 Hz higher octaves were chosen rather than the fundamental frequency. The sound samples used for the sound stimuli were recorded and then looped and projected in mathematically defined GS shapes and Stereo positioning (control). The same sound sample loop was used in all sound stimuli. Each sound stimulus was played for an epoch of 5 minutes. All samples were played on 4DSOUND using a spatial configuration of 48 omnidirectional loudspeakers placed above, below and around the subjects. All sound stimuli were played on the same output volume. Acoustic output level was adjusted to project the same loudness ensuring none of the GS shapes or Stereo was perceived louder to the listening subjects. Stereo sound stimulus was played in the same configuration as all GS shapes and according to the same sound projection characteristics. All sound stimuli were projected and recorded at <85 db. Presentation and order of sound stimuli randomly alternated between participants and was recorded on a double blind paradigm. The sound stimuli monitored were:

  0. Five minutes No Sound (Baseline)
  1. Five minutes Stereo sound (Control)
  2. Five minutes Pyramid GS
  3. Five minutes Sphere GS
  4. Five minutes Cube GS

Max dBA levels in dBFS and recordings of the presented stimuli are available in Supplementary 1.

### 2.3. Geometric Sound Shapes

All GS and stereo positioning were constructed in the same ratio with identical diameter and played on the same speaker configuration space. All GS shapes were constructed in relation to each other, for instance the length of the square *L* in Cube GS is identical to the length of the Pyramid base, all shapes are constructed with the same diameter.

The calculations for the GS shapes are as follows:

#### 2.3.1. Pyramid

The Pyramid is based on the (inverse) Golden Ratio (ratio = 1/0 : 618 = 1.618), which dictates its ratio and hence steepness. Pyramid base Length: *L* = 8 m. Height Pyramid: *H* = ratio *∗* *L* = 4,944 m. The Pyramid was decomposed into three (square) horizontal slices, from its base to its top (point) via an intermediary level, with the following coordinates:

1. Base: *L*1 = 8 m center is situated at (0, 0, 0)
2. Intermediary: *L*2 = 4,944 m center is situated at (0, 0, 1.889)
3. Top: *L*3 = 0 m, *y*3 = 4,944 m center is situated at (0, 0, 4.944)

#### 2.3.2. Cube

Sides of Cube = 8 m centered in space.

#### 2.3.3. Sphere

The Sphere is defined according to a ratio, such that the cube and the pyramid are inscribed in a sphere. The diameter *D* of the Sphere is computed according to the base length *L* of the cube and the pyramid:

(1) $$\begin{array}{c} D=\left(1+0.273\right)*L=10.184\;m. \end{array}$$

### 2.4. Sound Environment

Experiments were conducted on 4DSOUND [117] at MONOM Center for Spatial Sound Berlin. 4DSOUND technology integrates hardware and software systems to provide fully omnidirectional sound environments. The 57 channels 4DSOUND setup in MONOM studio consisted of 48 omnidirectional satellite Bloomline Acoustics OmniDrive Pro Mark II speakers in a 4 × 3 × 4 equally spaced grid, combined with 9 Bloomline Acoustics 4D OmniSub (Custom) subwoofers. The available spatialisation surface dimensions are 12 m × 12 m, and 4 m in height. In combination with the proprietary 4DSOUND software algorithms, this three-dimensional speaker configuration produces high definition sound spatialisation. The frequency range is 20 Hz to 20 kHz, and acoustic dynamics up to 116 dB. The sound output of each loudspeaker was determined by a computer-generated rated sound field consisting of virtual point-specific audio components and a panning algorithm using the 4DSOUND v1.3 software.

### 2.5. EEG

#### 2.5.1. Data Recording

**Figure 2:**
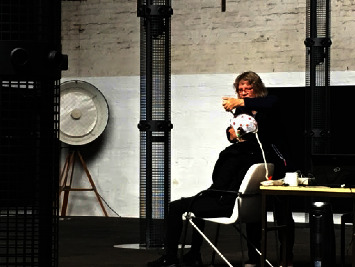
EX1 EEG measurements at MONOM center for spatial sound Berlin. The omnidirectional speakers are placed in the observed black columns. Each column holds 3 speakers: at ground level, top (4 m), and middle.

In EX1, data were collected and analyzed by neuroscientists from the BioKeshev Institute (IL; Figure 2).

In EX2, data were collected by neuroscientists from the Institut für EEG-Neurofeedback and the Peak Brain Institute (DE). In EX2, spectral and functional connectivity analysis as well as the statistical analysis of EEG results from EX1 and EX2 was conducted at the MEG-Center, University of Tübingen (DE). Participants' EEG was recorded in the standard 10/20 system. EEG was recorded continuously with a sampling rate of 256 Hz. Each sound stimuli was monitored for an epoch of 5 minutes. In EX1 the last minute of each epoch was analyzed and participants with noisy artifacts were removed from the analysis paradigm, for technical reasons only 10 participants were monitored for Pyramid GS. In EX2, subjects with noisy artifacts were removed from the analysis paradigm of brain waves power amplitude. For the connectivity analysis, segmented data of 500 ms duration each were visually inspected and epoques contaminated by artifacts were discarded. Using this procedure, all subjects could be considered for brain connectivity analysis.

#### 2.5.2. Spectral Power and Connectivity Analysis

Investigating the functional connectivity of brain activity by means of coherency, EEG data were analyzed using MATLAB (MathWorks Inc., Natick, Massachusetts USA), NBS Connectome [122], and FieldTrip, an open-source MATLAB toolbox specifically developed for the analysis of electrophysiological data [123].

After offset removal and high-pass filtering of the EEG data at 1 Hz data were segmented in junks of 500 ms. Eye and heart artifacts were removed using an Independent Component Analysis (ICA). In the subsequent frequency analysis a multitaper approach, with Discrete Prolate Spheroidal Sequences (DPSS) as tapers with a smoothing window of ±4 Hz was used. The Fourier spectrum of the signal was determined, selecting for frequencies ranging from 2 to 64 Hz, in steps of 2 Hz. The power in the Delta (1–4 Hz), Theta (5–8 Hz), Alpha (9–13 Hz), Beta-1 (14–20 Hz), Beta-2 (21–35 Hz), lower Gamma (36–48 Hz) and higher Gamma (52–75 Hz) frequency bands were calculated by averaging the power across frequency bins. The Gamma band was split into a lower and a higher band to avoid any power line artifacts at 50 Hz. Since the topographies of the lower and higher Gamma band activity were very similar, we collapsed both frequency bands. Connectivity analysis was performed on *n* trials at the sensor level, using the absolute part of imaginary coherency as measure of functional connectivity.

(2) $$\begin{array}{c} c_{ij}\left(f\right)=\left|\text{Imag}\left(\frac{\overset{n}{\underset{k=1}{\sum }}F_{ik}\left(f\right)F_{jk}^{*}\left(f\right)}{\sqrt{\overset{n}{\underset{k=1}{\sum }}F_{ik}\left(f\right)F_{ik}^{*}\left(f\right)\overset{n}{\underset{k=1}{\sum }}F_{jk}\left(f\right)F_{jk}^{*}\left(f\right)}}\right)\right|. \end{array}$$

With ∑_*k*=1_^*n*^*F*_*ik*_(*f*)*F*_*jk*_^*∗*^(*f*) and ∑_*k*=1_^*n*^*F*_*ik*_(*f*)*F*_*ik*_^*∗*^(*f*) being the cross and the auto spectra, respectively, of the EEG activity *F*_*ik*_(*f*) recorded in trial *k* at channels *i* and *j*. The absolute part of the imaginary part of coherency (Imag(.)) was chosen in order to avoid spurious connectivities due to the spreading of the signal to neighboring electrodes [124]. Given the electrodes, the output of the connectivity analysis was a *n* × *n*-connectivity matrix (19 × 19), quantifying the level of interaction between all pairs of EEG sensors.

#### 2.5.3. Network Statistical Analysis

NBS connectome [122] has been used to perform network statistical analyses, applying the cluster-based nonparametric method implemented in the network-based statistic (NBS) software [122]. A nonparametric way of testing was chosen due to the nonnormally distributed values of coherency. The cluster-based approach was chosen to avoid exhaustive multiple testing. Connectivity matrices were used as input for the analysis. For the statistical analyses in NBS, design matrices, and contrast vectors were defined in terms of a general linear model (GLM).

The Network-based statistic method involved four different steps. First, it runs a massive univariate test to define a test statistic value for each connection. Second, it compares the t-statistic of each connection, with the previously defined threshold set to *t* > 3.1. To capture medium to large effects of changes in connectivity, an effect size of 0.6 was considered relevant. With a sample size of 30 participants and using a conservative estimate, this effect size corresponds to a t-threshold of 3.1. Then, the presence of any topological clusters among the sets of supra-threshold connections is selected. To this end, significant connections that shared common nodes were summarized in individual clusters. Finally, to run a second level statistical testing comparing the differences of connectivity patterns across conditions, connectivity data of individual subjects were randomly assigned to the conditions to be compared. A total of 5000 permutations were performed in each permutation *t*. The size of the largest cluster, expressed in a one-sided FWER-corrected *p* value [125], is recorded. Permutations provide an empirical null distribution for the size of the largest cluster. For the statistical analysis pairwise comparisons between conditions were carried out.

### 2.6. Blood Pressure and Heart Rate

20 participants in EX1 were monitored by a trained nurse for their blood pressure and HR at baseline prior to exposure to the sound stimuli and immediately after each sound stimulus.

### 2.7. Behavioral Response and Questionnaires

In EX1, participants were required to answer Beck Depression Inventory (BDI) before enrolling in the experiment. Participants also answered a custom questionnaire composed by the researchers, to monitor their well-being and emotional response before and immediately after enrolling in the experiment (To view the full custom questionnaire, please see Supplementary 2). In EX2, participants were required to answer the Multidimensional Mood Questionnaire (MDMQ) and the same custom questionnaire as in EX1 before and immediately after enrolling in the experiment. BECK and the MDMQ are verified questionnaires and the custom questionnaire provided additional indication on the effect of the sound stimuli on participants without impinging the procedure. All questionnaires were administered in the same order both before and after the experiment. Participants were given the option to provide additional written testimonials of their experience.

### 2.8. Statistical Analysis

Statistical analysis of results of all questionnaires was conducted on SPSS 25.0 using paired *t*-test. Statistics were conducted for each experiment separately and the average response to both experiments was also computed. Not all items were filled in by all participants thus some were disqualified from being considered for the statistical analysis of results. Similarly, ANS markers and EEG parameters were compared between different GS conditions using paired *t*-tests; shape and sex differences for different GS conditions were studied using *t*-tests for independent samples. Significance of interactions between factors were tested by subjecting physiological values and individual characteristics and to multi-factor ANOVAs. By averaging the power across electrodes, a global power estimate was obtained for each GS type and each frequency band. To test statistical significance of the differences in power topography between the GS types, stereo and the baseline, cluster-based permutation statistics was used.

### 2.9. CymaScope

The CymaScope instrument makes sound visible by transforming audio input periodicities to water wavelet periodicities, the latter known as Faraday Waves or cymatic phenomena. A stereo recording from the center of the GS shape, where participants were placed, was recorded using Zoom H4n Pro with an angle of 120 degrees under the same sound projection conditions. Recordings were conducted at the same location and under identical conditions as EX1 and EX2. The recordings were sent to Cymascope laboratory in a stereo 24Bit, 48,000HR Wave format for analysis. Within the Cymascope instrument, the fused-quartz water-filled cuvette is direct-coupled to a Voice Coil Motor (VCM) with vertically driven piston. Electronic filtering is applied in the signal path to ensure that resonances, inherent in the VCM-cuvette assembly, are negated, resulting in a flat response characteristic curve. All samples were observed via direct ocular viewing, and then photographed using a Canon EOS 5D Mark camera and analyzed under identical conditions. The sound stimuli samples, as recorded and analyzed on the Cymascope instrument, are available at Supplementary 1.

## 3. Results

### 3.1. Physiological Response

#### 3.1.1. Brainwave Power Amplitude, Topology, and Connectivity Patterns (EX1 and EX2)

**Figure 3:**
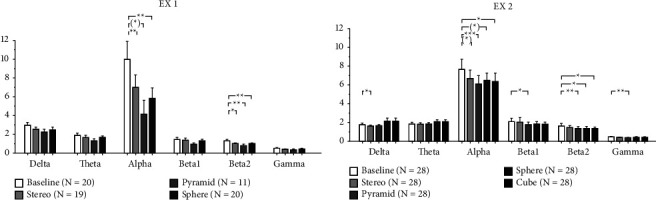
EEG-power in EX1 and EX2. Error bars represent the standard error of the mean. Significance level: ^*∗*^0.01 < *p* ≤ 0.05; ^*∗∗*^0.001 < *p* ≤ 0.01; ^*∗∗∗*^*p* ≤ 0.001; trend: (^*∗*^) 0.05 < *p* ≤ 0.1. (a) EX1 EEG power amplitude values alpha: baseline-stereo *p* ≤ 0.009, baseline-pyramide *p* ≤ 0.051, baseline-sphere *p* ≤ 0.002; beta2: baseline-stereo *p* ≤ 0.020, baseline-pyramide *p* ≤ 0.005, baseline-sphere *p* ≤ 0.005. (b) EX2 EEG power amplitude values. Delta: baseline-stereo *p* ≤ 0.038; alpha: baseline-pyramid *p* ≤ 0.001, baseline-sphere *p* ≤ 0.059, baseline-cube *p* ≤ 0.022, baseline-stereo *p* ≤ 0.061; beta1: baseline-pyramid *p* ≤ 0.046; beta2: baseline-pyramid *p* ≤ 0.009, baseline-sphere *p* ≤ 0.037, baseline-cube *p* ≤ 0.022; gamma: baseline-pyramid *p* ≤ 0.008.

A significant decrease in Alpha and Beta2 power amplitude was obtained during exposure to the different sound stimuli in both experiments, primarily by GS. Pyramid GS generated the most substantial power decrease across most frequency bands (Figure 3, Table 1).

In EX1, Alpha power significantly decreased between Baseline (9.995 ± 1.886 *μ*V^2^/Hz)-Sphere GS (5.857 ± 1.095 *μ*V^2^/Hz; *p* ≤ 0.002), and Baseline-Stereo (7.034 ± 1.264 *μ*V^2^/Hz; *p* ≤ 0.009). A trend towards significance was observed during exposure to Pyramid GS (4.155 ± 1.467 *μ*V^2^/Hz; *p* ≤ 0.051). Pyramid GS mean value analysis indicates a highly significant decrease which cannot be concluded due to the smaller sample size. Beta2 significantly decreased between Baseline (1.288 ± 0.157 *μ*V^2^/Hz) and all sound stimuli: Baseline-Pyramide (0.834 ± 0.145 *μ*V^2^/Hz; *p* ≤ 0.005), Baseline-Sphere (0.985 ± 0.084 *μ*V^2^/Hz; *p* ≤ 0.005), and Baseline-Stereo (1.004 ± 0.096 *μ*V^2^/Hz; *p* ≤ 0.020) (Figure 3(a)).

In EX2, which examined the response to an additional GS shape, a significant decrease in power was obtained during exposure to the different sound stimuli across most frequency bands. Highly significant results were obtained mainly during exposure to GS and predominantly Pyramid GS. In the Delta band, there was a significant decrease in Baseline (1.765 ± 0.141 *μ*V^2^/Hz)-Stereo (1.612 ± 0.116 *μ*V^2^/Hz; *p* ≤ 0.038). In the Alpha band, there was a highly significant decrease in Baseline (7.648 ± 1.024 *μ*V^2^/Hz)-Pyramid (6.100 ± 0.878 *μ*V^2^/Hz *μ*V^2^/Hz; *p* ≤ 0.001); significant decrease in Baseline-Cube (6.335 ± 0.887 *μ*V^2^/Hz; *p* ≤ 0.022); Trend towards significance in Baseline-Sphere (6.478 ± 0.762; *p* ≤ 0.059) and Baseline-Stereo (6.669 ± 0.902 *μ*V^2^/Hz; *p* ≤ 0.061). In the Beta1 band, there was a significant decrease Baseline (2.069 ± 0.380 *μ*V^2^/Hz)-Pyramid (1.765 ± 0.256 *μ*V^2^/Hz; *p* ≤ 0.046). In the Beta2 band: significant decrease Baseline (1.621 ± 0.229 *μ*V^2^/Hz)-Pyramid (1.378 ± 0.186 *μ*V^2^/Hz; *p* ≤ 0.009), Baseline-Sphere (1.378 ± 0.167 *μ*V^2^/Hz; *p* ≤ 0.037) and Baseline-Cube (1.365 ± 0.165 *μ*V^2^/Hz; *p* ≤ 0.022). In the Gamma band, there was a significant decrease in Baseline (0.478 ± 0.069 *μ*V^2^/Hz)-Pyramid (0.386 ± 0.051 *μ*V^2^/Hz; *p* ≤ 0.008) (Figure 3(b)).

**Figure 4:**
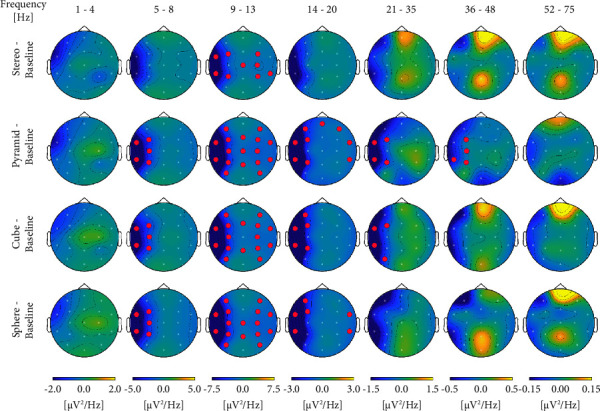
Significant results of topological analysis. Red spots represent areas of significant change in activity in the theta, alpha, beta1, beta 2, and gamma bands during exposure to different sound stimuli compared to baseline (*N* = 30).

Integrated analysis of both experiments reveals that significant results are obtained across most measured frequency bands, primarily during exposure to GS. Highly significant results are obtained in the Alpha, Beta2, and Gamma bands exclusively during exposure to GS. Results of the comparative analysis across different conditions remained consistent between EX1 and EX2, displaying parallel trends. Cube GS, which was solely monitored in EX2, is not present in this analysis. In the Delta band, there was a significant decrease between Baseline (2.278 ± 0.171 *μ*V^2^/Hz)-Stereo (1.979 ± 0.135 *μ*V^2^/Hz; *p* ≤ 0.015) and Baseline-Pyramide GS (1.831 ± 0.121 *μ*V^2^/Hz; *p* ≤ 0.024). In the Alpha band, there was a highly significant decrease between Baseline (8.626 ± 0.989 *μ*V^2^/Hz)-Pyramide GS (5.552 ± 0.757 *μ*V^2^/Hz; *p* ≤ 0.001), Baseline-Sphere GS (6.219 ± 0.631 *μ*V^2^/Hz; *p* ≤ 0.001), and a significant decrease between Baseline-Stereo (6.817 ± 0.733 *μ*V^2^/Hz *μ*V^2^/Hz; *p* ≤ 0.002). In the Beta1 band, there was a significant decrease between Baseline (1.820 ± 0.236 *μ*V^2^/Hz)-Pyramide GS (1.536 ± 0.197 *μ*V^2^/Hz; *p* ≤ 0.023). In the Beta2 band: highly significant decrease between Baseline (1.482 ± 0.149 *μ*V^2^/Hz)-Pyramide GS (1.224 ± 0.144 *μ*V^2^/Hz; *p* ≤ 0.001), Baseline-Sphere GS (1.214 ± 0.107 *μ*V^2^/Hz; *p* ≤ 0.001) and a significant decrease between Baseline-Stereo (1.261 ± 0.136 *μ*V^2^/Hz; *p* ≤ 0.004). In the Gamma band, there was a highly significant decrease between Baseline (0.492 ± 0.046 *μ*V^2^/Hz)-Pyramide GS (0.382 ± 0.040 *μ*V^2^/Hz; *p* ≤ 0.001), and a significant decrease between Baseline-Stereo (0.403 ± 0.032 *μ*V^2^/Hz; *p* ≤ 0.009) (Table 1). Further computation of results was applied using unpaired comparisons with factor sex. Analysis revealed statistical significant results predominantly during exposure to Pyramide GS. Results indicate that female participants continuously present more power and are more reactive to GS stimuli in most frequency bands compared to Baseline and Stereo. Importantly, results vary during exposure to the different sound stimuli, indicating the response to be sex and shape dependent. In the Delta band in Pyramid GS (F:2.092 ± 0.199, M:1.608 ± 0.131 *μ*V^2^/Hz; *p* ≤ 0.044). In the Beta1 band, in Sphere GS (F: 1.981 ± 0.320, M: 1.297 ± 0.111 *μ*V^2^/Hz; *p* ≤ 0.036). In the Beta2 band, in Pyramid GS (F: 1.537 ± 0.272, M: 0.956 ± 0.107 *μ*V^2^/Hz; *p* ≤ 0.042). In the Gamma band, in Pyramid GS (F: 0.472 ± 0.066, M: 0.306 ± 0.043 *μ*V^2^/Hz; *p* ≤ 0.036) and in Stereo (F: 0.508 ± 0.052, M: 0.310 ± 0.029 *μ*V^2^/Hz; *p* ≤ 0.002). Analysis of EX2 recordings extended to topology and connectivity patterns to provide supplementary insights into power amplitude results. The findings revealed significant topological changes primarily during exposure to all GS projections. Notably, Pyramid, Cube, and Sphere GS demonstrated symmetric Alpha topography compared to Baseline and Stereo. Significant topological changes, predominantly in the left hemisphere, were additionally detected in the Theta, Beta1, Beta2, and Gamma bands during exposure to all GS projections and not Stereo. In the Gamma band changes were present exclusively in Pyramid and Cube GS. Pyramid GS displayed the most significant topological activity across the widest frequency range (Figure 4).

**Figure 5:**
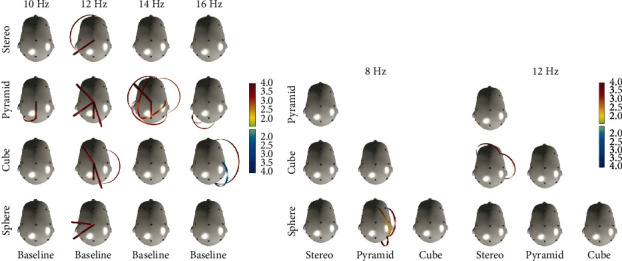
Significant results of connectivity patterns in the range of 10 Hz to 16 Hz area. (a) Significant results of different connectivity patterns are mostly present at 12 Hz between baseline, GS shapes (pyramid, cube, sphere) and stereo (control). Red lines indicate significantly enhanced connectivity in exposure to sound stimuli; blue lines indicate significantly enhanced connectivity in baseline. Different connectivity patterns are observed mostly with pyramid GS in the range between 10 Hz and 16 Hz (*N* = 30). (b) Comparison of connectivity patterns between all sound stimuli. GS shapes sphere-pyramid at 8 Hz and cube-stereo at 12 Hz (*N* = 30).

Connectivity patterns analysis further reinforces observed trends in brainwave power and topology differences between GS and the Stereo. Notably, the analysis reveals that an identical sound frequency sample elicits varying and distinct connectivity patterns during exposure to each sound stimulus projection, particularly in the case of GS. Importantly, as values of power amplitude significantly decrease across frequency bands, an independent increase in connectivity patterns is obtained, rendering it as a noteworthy result. Concentrating on statistically significant connectivity clusters only, significant and varied connectivity patterns are observed in the Alpha band, predominantly at 12 Hz, during exposure to all sound stimuli. Patterns of connectivity mostly involve the central/left electrodes. Further in the Alpha and Beta1 bands, different connectivity patterns are observed in Pyramid GS compared to Baseline between 10 Hz and 14 Hz, and in Cube GS at 16 Hz (Figure 5(a)). Significant patterns are additionally observed between Sphere GS and Pyramid GS at 8 Hz and between Cube GS and Stereo at 12 Hz (Figure 5(b)). Other significant connectivity patterns are noted at more sephoradic frequencies in the Beta2 and Gamma bands, primarily during exposure to GS and predominantly Pyramid GS; for more information please see Supplementary 3. Notably, results on all EEG analysis parameters-topologic power amplitude and connectivity patterns are consistent with respect to the larger effect of GS compared to Stereo, predominantly in the Alpha band. Significant effects are additionally noted in the Delta, Theta, Beta1, Beta2 and Gamma bands. Across analysis paradigms Pyramid GS demonstrated the most significant effect.

#### 3.1.2. Heart Rate (EX1)

General HR between all participants decreased during EX1 from a mean of 73 Beats Per Minute (BPM) at Baseline to a mean of 69 BPM after Sphere GS and Stereo (control), and 67 BPM after Pyramid GS. Statistical significance is obtained between Baseline (73.263 ± 1.837)-Sphere GS (68.842 ± 1.924; *p* ≤ 0.012) and between Baseline-Stereo (68.842 ± 1.664; *p* ≤ 0.003). Computation of mean results for male and female participants indicates that male participants show enhanced reaction to GS compared to Stereo. Female participants were equally affected by all sound stimuli (Table 2).

#### 3.1.3. Blood Pressure (EX1)

Mean blood pressure of participants decreased from 120/80 at Baseline, which is considered elevated blood pressure according to the American Heart Association, to 113/78 following Stereo, 112/76 following Sphere GS and 111/77 following Pyramid GS, all considered normal. Upon further computation, significant results were independently obtained for both Shape and Sex factors concerning systolic and diastolic pressure. Shape-dependent results demonstrate a highly significant decrease in systolic blood pressure between Baseline (120.263 ± 2.492)-Sphere GS (111.579 ± 2.538; *p* ≤ 0.001) and Baseline-Stereo (113.158 ± 2.571; *p* ≤ 0.001) and a significant decrease between Baseline-Pyramid GS (111.111 ± 2.736; *p* ≤ 0.012). A significant decrease in diastolic blood pressure was only obtained between Baseline (80.263 ± 1.455)-Sphere GS (76.053 ± 1.603; *p* ≤ 0.002). Significant sex effects were obtained exclusively in systolic blood pressure. In general, female participants exhibited a lower blood pressure than males: Baseline (F: 113.125 ± 1.875, M: 125.455 ± 3.334; *p* ≤ 0.009), Stereo (F: 106.875 ± 2.662, M: 117.727 ± 3.46; *p* ≤ 0.032), Sphere (F: 105.000 ± 2.315, M: 116.364 ± 3.44; *p* ≤ 0.022) (Table 3). No significant interactions were found between shape and sex factors.

### 3.2. Behavioral Response

#### 3.2.1. Questionnaires and Experiential Testimonials of Participants (EX1 and EX2)

**Figure 6:**
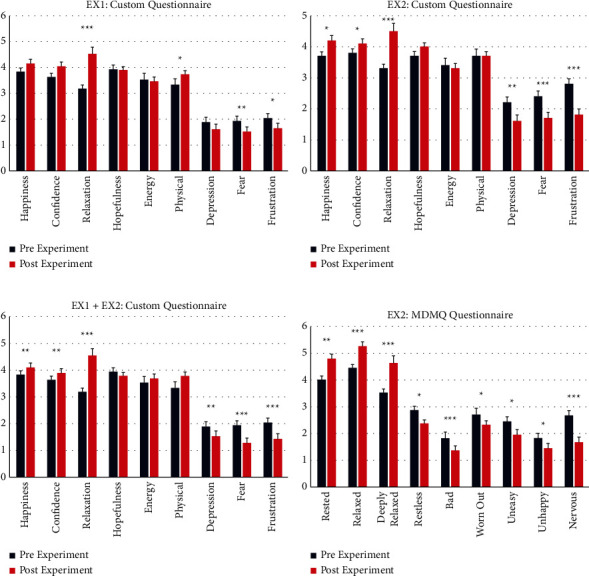
Significant results to behavioral questionnaires. (a–c) The results of all items in the custom questionnaire. (d) Statistically significant results of 9 items out of an overall of 30 items in the MDMQ questionnaire. Significance level ^*∗*^0.01 < *p* ≤ 0.05; ^*∗∗*^0.001 < *p* ≤ 0.01; ^*∗∗∗*^*p* ≤ 0.001. (a): EX1 results to custom questionnaire, (*N* = 20). Relaxation *p* ≤ 0.001, physical *p* ≤ 0.025, fear *p* ≤ 0.008, frustration *p* ≤ 0.014. (b): EX2 results to custom questionnaire, (*N* = 28). Happiness *p* ≤ 0.011, confidence *p* ≤ 0.015, relaxation *p* ≤ 0.001, depression *p* ≤ 0.007, fear *p* ≤ 0.001, frustration *p* ≤ 0.001. (c): EX1 and EX2 results to the custom questionnaire, (*N* = 48). Happiness *p* ≤ 0.004, confidence *p* ≤ 0.002, depression *p* ≤ 0.002, fear *p* ≤ 0.001, relaxation *p* ≤ 0.001, frustration *p* ≤ 0.001. (d): EX2 statistically significant results to MDMQ questionnaire, (*N* = 26). Rested *p* ≤ 0.002, relaxed *p* ≤ 0.001, deeply relaxed *p* ≤ 0.001, restless *p* ≤ 0.045, bad *p* ≤ 0.001, worn out *p* ≤ 0.030, uneasy *p* ≤ 0.025, unhappy *p* ≤ 0.048, nervous *p* ≤ 0.001.

In EX1 custom questionnaire, highly significant results were obtained in increased sense of relaxation (*p* ≤ 0.001); significant results were obtained in improved physical sense (*p* ≤ 0.025) and decreased sense of fear (*p* ≤ 0.008) and frustration (*p* ≤ 0.014) (Figure 6(a)).

In EX2, highly significant results were obtained in increased sense of relaxation (*p* ≤ 0.001), and decreased sense of fear (*p* ≤ 0.001) and frustration (*p* ≤ 0.001). Significant results were obtained in increased sense of happiness (*p* ≤ 0.011) and confidence (*p* ≤ 0.015) and decreased sense of depression (*p* ≤ 0.007) (Figure 6(b)). Integrated analysis of results from both experiments shows highly significant results in increased sense of relaxation (*p* ≤ 0.001) and decreased sense of fear (*p* ≤ 0.001) and frustration (*p* ≤ 0.001). Significant results are found in increased sense of happiness (*p* ≤ 0.004) and confidence (*p* ≤ 0.002) and decreased sense of depression (*p* ≤ 0.002) (Figure 6(c)). Notably, the positive trend towards improved general wellbeing is maintained in items that did not generate significant statistical results (Figures 6(a)–6(c)). In EX2 MDMQ questionnaire, highly significant results were obtained in increased sense of Relaxation (*p* ≤ 0.001) and Deep Relaxation (*p* ≤ 0.001), and decreased sense of Nervous (*p* ≤ 0.001) and Bad (*p* ≤ 0.001) feelings. Significant results were obtained in increased sense of feeling Rested (*p* ≤ 0.002), and decreased sense of feeling Worn Out (*p* ≤ 0.030), Uneasy (*p* ≤ 0.025), Restless (*p* ≤ 0.045) and Unhappy (*p* ≤ 0.048) (Figure 6(d)).

Participants had the freedom to elaborate on all items in the custom questionnaire and provide testimonials. Testimonials align with the quantitative responses gathered from the MDMQ and custom questionnaires, as well as ANS measurements. 95.8% of participants reported the overall experience was positive, uplifting, and beneficial. Participants pointed out a combination of positive sensations such as a sense of deep relaxation, enhanced sense of focus, improved sense of self-esteem, confidence, calmness, hopefulness, and vitality, improved sense of physical feeling, enhanced imagery including elaborated images, colors and memories. Participants largely refer to a feeling or sensation similar to meditation. Some examples of quotes from participants include: *“I feel happy and relaxed, much calmer after than before,” “Noticeable less agitated by everything,” “I feel more relieved, also physically and possibly more elastic,” “Sounds helped me calm and see the good rather than the bad.”.* (For documentation of all testimonials please see Supplementary 4).

### 3.3. Faraday Waves Pattern Morphology

**Figure 7:**
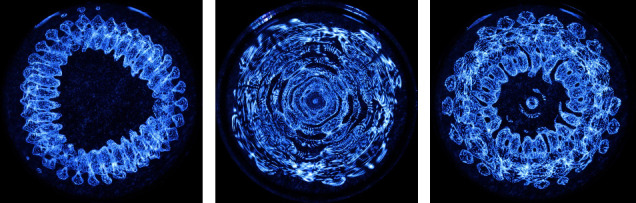
Faraday wave patterns morphology as documented using the CymaScope instrument. (a) Pyramid GS projection recording resultant faraday wave pattern. (b) Cube GS projection recording resultant faraday wave pattern. (c) Sphere GS projection recording resultant faraday wave pattern.

Analysis of the resulting Faraday wave patterns morphology revealed a correlation between the excitation GS projection recording and the resultant Faraday wave pattern. Specifically, Pyramid GS generates a triangular pattern, Cube GS generates a square pattern, and Sphere GS generates a circular pattern (Figures 7(a)–7(c)). Notably, all sound stimuli play the exact same sound sample and frequency combination (272.2 Hz & 544.4 Hz) played in an identical loop, duration, loudness, and measured under identical conditions. Faraday wave observations indicate a similar trend to the one observed in EEG analysis, demonstrating each GS shape projection generates specific patterns independently of frequency.

## 4. Discussion

This study investigated the influence of Geometric Sound (GS) on brain waves activity patterns, HR, blood pressure, emotional well-being, and Faraday wave pattern morphology. Significant variations in brainwave activity patterns were observed in all measured frequency bands during exposure to GS compared to Baseline and Stereo (control) condition. A predominant effect was noted in the Alpha band, characterized by a significant decrease in power, symmetric topology, and discrete connectivity patterns across the widest cluster. Significant results in power amplitude, topology, and connectivity patterns were additionally observed in the Delta, Theta, Beta1, Beta2, and Gamma bands. Pyramide GS demonstrated the most significant results and largest effect across analysis paradigms and frequency bands. Power amplitude results computation from both experiments validates that comparative analysis trends remain consistent between EX1 and EX2. Furthermore, the observed decrease in power amplitude validates that connectivity patterns are of high significance. Interestingly, in the Alpha band connectivity, patterns are more predominant in the left and central regions while in the Beta2 and Gamma bands they are more prevalent in the right and central regions. According to behavioral reports and ANS measurements, GS is prone to positively reduce HR and blood pressure, increase relaxation, positive feelings and stress-related coping mechanisms while reducing negative feelings. All monitored biomarkers indicate that autonomic response varies predominantly according to factor Shape and to some degree to factor Sex. Across all monitored biomarkers GS consistently yielded more significant results when compared to the Stereo condition (control). Faraday wave analysis exhibited discrete morphology patterns correlating to the excitation GS shape recording. These observations correspond to EEG and ANS analysis of discrete and shape-dependent activity patterns, possibly suggesting a causal relationship. Our hypothesis suggests that the mechanism leading to the enhanced effect of GS compared to Stereo is derived from the geometric-mathematical information encoded within the sound projection and is based on mathematical rules. We further suggest this process results in a feedback loop and a “mirroring” effect, implying geometric-mathematical information could be applied externally and employed internally to bring natural systems into coherence. We speculate that the heightened results observed following exposure to Pyramid GS may be attributed to its exclusive incorporation of the Phi ratio.

Results of decreased Alpha power in conjunction with behavioral and ANS response are in agreement with studies monitoring the effect of relaxing sound on brain activity and general well-being. High resolution sound stimuli was reported to enhance relaxation compared to low resolution sound stimuli while showing decrease in Alpha activity [63]. A decrease in Alpha and Beta2 activity was reported while monitoring relaxation effects of listening to monochord compared to progressive muscle relaxation tapes of the same length and sonic characteristics during chemotherapy. The researches indicate their results correlate with a significant improvement in participants' physical and psychological states as well as in their state of anxiety [47, 126]. Lower levels of Alpha activity in the left front central region, as demonstrated here, were associated with higher levels of self-acceptance, environmental mastery, personal growth and psychological well-being [127]. Physically, studies indicate that Alpha decrease could suggest a positive effect on cardiovascular and respiratory systems in accordance with mood induction [128] and higher levels of blood oxygenation [129]. Results are in line with research showing reduced HR, reduced systolic and diastolic blood pressure along with improved pain endurance following exposure to music therapy [130]. Alpha power was shown to decrease during arithmetic tasks compared to purely mental tasks while being immersed in a Virtual Reality (VR) experience, suggesting by the authors that attention is being imposed inwards [131]. EEG results in conjunction with participants' testimonials, ANS markers, and behavioral response imply that participants were in a state of enhanced concentration [132] and relaxed alertness, also referred to as orchestrated immersion, which is considered the optimal condition for learning as focused attention is inhibited [133, 134]. We suggest that spatial information is derived from the geometric and mathematical attributes of sound by the brain. We further suggest that the mathematical component of GS may support analytical and logical thinking, typically associated with left hemisphere processing, while its sonic and melodic aspects may support holistic and creative thinking, usually attributed to right hemisphere processing. This interpretation is supported by localization of connectivity patterns during exposure to GS across left and right hemispheres.

Importantly, brain waves are known to entrain to a sound frequency, or combination of frequencies (interval) [38–41]. This study indicates that brain waves are affected differently not only by sound frequencies but also by the spatial properties of sound propagation, i.e. projection. Results reveal distinct patterns of power amplitude, topology, and connectivity during exposure to the different sound stimuli Pyramid, Cube, Sphere GS and Stereo (control)-all playing an identical sound sample of an octave interval between 272.2 Hz and 544.4 Hz. Discrete significant changes were observed across analysis parameters in all monitored brainwave frequency bands. Notably, at this point we cannot deduce whether the observed patterns in brainwave activity are purely a result of GS spatial projection, its harmonic spectra, or a combination of both factors. It is plausible that different intervals, frequency ranges and individual frequencies will produce alternative patterns across brainwave bands and brain regions. While the effect of immersion in GS was largely compared by participants to meditation, brain activity patterns differ. Meditation and mindfulness are commonly related to increased activity in the Alpha and Theta bands, [135, 136] which was not observed in this study. However, it has been found that different types of meditation as well as different types of neurofeedback result in a variance of brainwave activity patterns [137, 138]. It is reasonable to assume that GS activates the brain differently compared to methods related to silence, while achieving similar results of increased relaxation, sense of focus, self-awareness, positive emotional and physical function [30, 61–65]. We speculate that the positive effect of relaxation following regular meditation and mindfulness practice, also found to result in improved immune function [100], would sustain with continued exposure to GS.

This preliminary study targeted healthy young adults to demonstrate potential effects in mundane situations and establish a safety baseline before considering studies on populations with more acute conditions. For example, ADHD and Major Depressive Disorder (MDD) are associated with distinct neurologic and emotional patterns. Results demonstrate that topologic response to GS is leading to decreased activity in the left hemisphere and could benefit ADHD challenges linked to increased left hemisphere activity in frontal and temporal regions during intellectual stress [139, 140]. Further, MDD is associated with a decrease in Alpha symmetry [135, 136]; Exposure to all GS shapes demonstrated Alpha symmetry, suggesting potential benefits for MDD rehabilitation.

Furthermore, we posit that the findings presented here in brainwave activity patterns, ANS and behavioral measurements, may suggest a novel approach for neurotrauma rehabilitation which could support benefits in desired plasticity. Importantly, Brain plasticity and Hebbian Learning [141] are crucial for neurotrauma rehabilitation [45, 46, 137] and are used in non-invasive practices such as brain-computer interface (BCI) and neurofeedback processes to model and ignite non-functional areas into activity [138, 139]. Research found significant behavioral and structural impairments improvements post-neurotrauma and ADHD following such non-invasive approaches [101, 142], further underscoring the role of plasticity in recovery [44–46]. Individuals recovering from neurotrauma, stemming from either Acquired Brain Injury (ABI) or Traumatic Brain Injury (TBI), frequently exhibit stress-related emotional deficits, agitation and depressive symptoms [143–145]. Biomedical theories suggest that sound and music can stimulate neuroplasticity and engage cortical regions, including those adjacent to the auditory cortex [145, 146]. In accordance, music interventions post-ABI and TBI were found to positively affect brain connectivity networks, mechanical function such as gait and coordination, improve social skills, communication and general well-being, improve mood disorders such as depression and irritability and improve general quality of life [45, 46, 145, 147–150]. Similar significant results between live and recorded music intervention sessions were reported following TBI on agitation and post-traumatic amnesia [145]. Post-TBI music intervention was reported to affect within and between network connectivity changes in cognitive networks, increased connectivity between frontal and parietal regions, increase gray matter volume in right inferior frontal gyrus, increase coupling in networks which are associated with TBI cognitive impairments, as well as between these networks and primary sensory networks. A shift towards decreased connectivity in networks typically hyperconnected following TBI was also reported [45]. Importantly, no harmful results were reported across studies monitoring various types of music interventions on neurotrauma [45, 145–149, 151, 152]. We posit that upon further investigation of exposure to GS, distinct connectivity patterns could be determined and utilized to establish plasticity in desired regions. GS proposes a method that does not require active participation and could support rehabilitation for consciously compromised conditions. Importantly, GS is indicated to incorporate brain plasticity and cognitive rehabilitation processes while being emotionally beneficial, as it integrates the merits of sound and music therapies, both found to be highly beneficial for neurotrauma rehabilitation.

Results of behavioral reports and ANS markers highlight the positive impact of sound medicine on emotional well-being and indicate it to be a valid non-invasive method to improve relaxation and relieve stress after a short exposure. Both the MDMQ and custom questionnaires demonstrate highly correlated patterns indicating improved emotional well-being following a 20 minutes exposure to the different sound stimuli. A highly significant increase was reported in sense of relaxation, deep relaxation and rested feelings along with a highly significant decrease in reported sense of nervousness, frustration, fear and bad feelings. Significant increase was reported in the sense of happiness and confidence along with a significant decrease in reported sense of depression, restlessness, worn out and uneasiness. General testimonials from participants indicate improved sense of emotional and physical well-being including improved sense of physical comfort, enhanced sense of focus, enhanced imagery including elaborated images, colors and memories, meditative feeling, and improved self-esteem.

While it is difficult to differentiate the effect between the sound stimuli by behavioral response, further information is indicated from EX1 ANS markers measurements. Discrete changes in HR and blood pressure were found to be shape dependent, and to a lesser extent sex dependent. A statistically significant shape-dependent decrease in HR was measured following exposure to Sphere and Stereo conditions, in systolic blood pressure following exposure to all sound stimuli, and in diastolic blood pressure exclusively following exposure to Sphere GS. Sex-dependent analysis further demonstrates discrete patterns of response in ANS measurements. Mean computation indicates male participants responded more favorably to GS compared to Stereo. Male participants had a mean HR of 72.7 BPM at Baseline and 70.1 BPM following exposure to Stereo, both considered average HR for this age group at rest. Following Sphere GS HR decreased to 69.4 BPM, and after Pyramid GS to 66 BPM, both considered good for resting conditions in this age group. Female participants had a mean HR of 74 BPM at Baseline, considered average for this age group at rest. After exposure to Stereo, HR decreased to 67 BPM, and following exposure to Sphere GS and Pyramid GS to 68 BPM, all considered excellent for resting conditions in this age group. Significant sex-dependent results in systolic blood pressure were found between Baseline and Sphere GS, Baseline and Stereo, as well as between Stereo and Sphere GS. Similarly to HR mean results, female participants are indicated to demonstrate a decrease in systolic and diastolic blood pressure following exposure to all sound stimuli, while male participants demonstrate a decrease in systolic and diastolic blood pressure exclusively following exposure to GS. This trend is not confirmed in the statistical analysis. However, given that males in this age group typically exhibit higher blood pressure compared to females, a separate analysis focusing on male participants revealed that exposure to Pyramid GS resulted in an enhanced decrease in systolic and diastolic blood pressure, aligning it more closely with the levels observed in female participants. We postulate that due to the smaller sample size exposed to Pyramid GS, no statistical significance was obtained (F: 108.333 ± 3.333, M: 112.500 ± 3.819, *p* ≤ 0.509). Notably, sex-dependent EEG results computation also revealed significant patterns primarily during exposure to Pyramide GS in the Delta, Beta2 and Gamma bands. Additional significant results were obtained during exposure to Sphere GS in the Beta1 band, and during exposure to Stereo in the Gamma band. Importantly, across both experiments the same trend is maintained in power between male and female participants. Female participants continuously demonstrate more power. Interestingly, there is no constant power ratio between male and female power values that could indicate an anatomical sex-dependent difference. Instead, the ratio between male and female power values varies depending on the stimulus, thus rendering it a reactive measurement to the exhibited stimulus rather than an attribute of an anatomical property. The underlying mechanism for the differential responses observed between male and female participants in EEG and ANS markers is still unclear. Importantly, due to the smaller sample size of participants and especially female participants exposed to Pyramid GS in EX1, female ANS analysis in response to this condition lacks empirical data and conclusions should be taken with care.

Notably, similarly to the consistent trend observed in EEG and ANS analysis, Faraday wave observations indicate that each GS shape projection results in a specific and correlating pattern. Faraday wave pattern morphology indicates that GS excitation generates a unique mirroring wave pattern to the excitation stimulus. Hence, identical sound frequency samples result in distinct Faraday wave patterns correlating with the spatial projection of sound. This result is novel in the field of sound induced Faraday waves, suggesting pattern morphology is affected by the spatial properties of sound, regardless of the monitored frequency and boundary condition of the observed medium. The effect shown on brainwave power amplitude, topology and connectivity patterns, ANS markers and Faraday waves pattern morphology suggest sound characteristics other than excitation frequency can result in varied response patterns. It is interesting to note that following an audible sound excitation the effect on cells is indicated to vary, influenced by waveform independently of frequency [153]. Furthermore, the documented images indicate the sound is indeed projected in its specified geometric shape, potentially serving as validation for the holographic principle of sound. The holographic principle of sound hypothesis suggests that sound fragments encapsulate its spectral and spatial information. Thus, even a mono or stereo recording of a 3D GS shape projection includes characteristics of its spatial properties projected in space [105]. We suggest this indication signifies a form of geometric synchronization, as previously proposed, potentially extending to other geometric substances such as molecules. For example, disrupted crystal structure of polluted water, or disrupted geometric molecule binding could potentially be restored to its coherent geometric structure through external geometric stimuli such as GS. In biology, neuroscience and behavioral design it has been shown that structure and function are intimately related-shape informs function and function determines form [13, 129, 154, 155]. It is probable that shape represents function and could possibly affect it. In a fractal syntropic system such as nature, including human physiology and psychology, shape's function informs and inevitably constantly imposes its characteristics on its surroundings. It is a feedback loop between developmental stages governed and tightly related to mathematics and geometry.

### 4.1. Study Limitations

Participants were recruited through an open call and hold relatively homogeneous characteristics. EEG results were collected by two different teams positioned slightly differently in the space. However, participants were placed in the same location in the space and of the sound projection. The stimuli were projected within <85 db and was manually mixed to the same perceived loudness; measured levels of SPL were not collected. Notably, spatial sound projections are indicated to result in significant comparative differences between measured SPL, thus the audial perceived loudness of the projection does not necessarily correlate to its SPL. Faraday Waves data were documented once; further observations should be conducted to verify the presented results. The effect of the sound stimuli frequency should be further investigated-it is unclear if presented results are solely due to the projected GS, the excitation frequencies or a combination of both. These aspects should be further investigated to promote understanding of sound frequencies and spatial sound on human physiology and Faraday waves pattern morphology. This research tested three GS shapes (Pyramid, Cube, and Sphere), further research should document the effect of more GS shapes and other mathematical sound attributes. Despite these limitations this is an important study demonstrating sound has distinct effects on biological mechanisms and can influence them.

## 5. Conclusion

Observations on nature posit that geometric symmetry is intertwined with effective energy distribution and coherence. Such indications form the foundation for the present study which sought to integrate spatial sound technology, geometry and sound medicine to regulate natural systems through the implementation of a geometric feedback loop. Significant changes and varying activity patterns in EEG recordings were observed on three analysis paradigms during exposure to Geometric Sound (GS) compared to Stereo (control) and Baseline in all monitored frequency bands. A clear tendency towards improved sense of relaxation, general well-being, enhanced positive feelings and decreased negative feelings was reported by participants. ANS parameters further indicate a clear tendency towards relaxation. Effects are primarily shape-dependent and to some extent sex-dependent, mechanisms for sex dependency patterns are not clear. The enhanced results of GS compared to Stereo, as well as distinctions in response to the studied GS shapes, were consistently observed across brainwave analysis paradigms, HR and blood pressure. GS effect on brainwave and ANS markers indicates each GS projection, hence mathematical information, is generating a unique connectivity pattern and physiologic response. Similarly, GS was shown to affect Faraday Waves pattern formation, indicating sound's spatial properties impact its pattern morphology.

Results suggest regulation of human physiology, psychology, and matter by means of sonic stimuli of a geometric and mathematical nature. We propose GS to be considered as a non-invasive rehabilitation method for different emotional and cognitive conditions such as ADHD, MDD and anxiety. We further suggest that the well-documented effect of music and sound interventions on neurotrauma rehabilitation, including mood disorders, limbic and physical function and neural plasticity, could be personally adjusted by using specific GS projections and their resultant discrete connectivity patterns to enhance desired plasticity. Further studies on GS should shed more light on the underlying mechanism resulting in these effects.

## Figures and Tables

**Table 1:** Integrated analysis of EEG-Power in EX1 and EX2.

EX1 & EX2 power amplitude			
Frequency band	df	*p*	*N*
*Delta*			
Baseline–pyramid	38	^*∗*^ ** 0.024**	39
Baseline–sphere	47	0.965	48
Baseline-cube	27	0.466	28
Baseline–stereo	46	^*∗*^ ** 0.015**	47

*Theta*			
Baseline–pyramid	38	0.213	39
Baseline–sphere	47	0.690	48
Baseline-cube	27	0.253	28
Baseline–stereo	46	0.275	47

*Alpha*			
Baseline–pyramid	38	^*∗∗∗*^ ** 0.001**	39
Baseline–sphere	47	^*∗∗∗*^ ** 0.001**	48
Baseline-cube	27	^*∗*^ ** 0.022**	28
Baseline–stereo	46	^*∗∗*^ ** 0.002**	47

*Beta1*			
Baseline–pyramid	38	^*∗∗*^ ** 0.023**	39
Baseline–sphere	47	0.056	48
Baseline-cube	27	0.223	28
Baseline–stereo	46	0.640	47

*Beta2*			
Baseline–pyramid	38	^*∗∗∗*^ ** 0.001**	39
Baseline–sphere	47	^*∗∗∗*^ ** 0.001**	48
Baseline-cube	27	^*∗*^ ** 0.022**	28
Baseline–stereo	46	^*∗∗*^ ** 0.004**	47

*Gamma*			
Baseline–pyramid	38	^*∗∗∗*^ ** 0.001**	39
Baseline–sphere	47	0.065	48
Baseline-cube	27	0.109	28
Baseline–stereo	46	^*∗∗*^ ** 0.009**	47

Significance level: ^*∗*^0.01 < *p* ≤ 0.05; ^*∗∗*^0.001 < *p* ≤ 0.01; ^*∗∗∗*^*p* ≤ 0.001. Results for cube GS are taken from EX2.

**Table 2:** EX1 Mean heart rate among participants following exposure to the different sound stimuli.

Heart beat (BPM)	Baseline	Sphere GS	Pyramid GS	Stereo (control)
General	73 BPM *N* = 19	69 BPM *N* = 19 ^*∗*^**p** ≤ 0.012	67 BPM *N* = 9	69 BPM *N* = 19 ^*∗∗*^**p** ≤ 0.003

Male	72.7 BPM *N* = 11 (Average)	69.4 BPM *N* = 11 (Good)	66 BPM *N* = 6 (Good)	70.1 BPM *N* = 11 (Average)

Female	74 BPM *N* = 8 (Average)	68 BPM *N* = 8 (Great)	68 BPM *N* = 3 (Great)	67 BPM *N* = 8 (Great)

Significance level: ^*∗*^0.01 < *p* ≤ 0.05; ^*∗∗*^0.001 < *p* ≤ 0.01; ^*∗∗∗*^*p* ≤ 0.001. In brackets are indications for heart rate condition in a resting state for the monitored sex and age group. A lower heart rate at rest implies more efficient heart function.

**Table 3:** EX1 shape-dependent and sex-dependent statistical results of systolic and diastolic blood pressure among participants following exposure to the different sound stimuli.

Condition	Main effect shape			Main effect sex		
	df	*F*	*p*	df	*F*	*p*
BP_sys baseline–stereo (*N* = 19)	1, 17	16.984	^*∗∗∗*^ ** 0.001**	1, 17	7.889	^*∗*^ ** 0.012**
BP_sys baseline–sphere (*N* = 19)	1, 17	22.487	^*∗∗∗*^ ** 0.001**	1, 17	8.822	^*∗∗*^ ** 0.008**
BP_sys baseline–pyramid (*N* = 9)	1, 7	11.200	^*∗*^ ** 0.012**	1, 7	2.223	0.1796
BP_sys stereo–sphere (*N* = 19)	1, 17	1.498	0.2377	1, 17	6.383	^*∗*^ ** 0.021**
BP_sys stereo–pyramid (*N* = 9)	1, 7	0.027	0.8731	1, 7	0.916	0.3703
BP_sys sphere–pyramid (*N* = 9)	1, 7	0.093	0.7689	1, 7	0.275	0.6165
BP_dia baseline–stereo (*N* = 19)	1, 17	2.075	0.1679	1, 17	1.908	0.1851
BP_dia baseline–sphere (*N* = 19)	1, 17	12.194	^*∗∗*^ ** 0.002**	1, 17	0.919	0.3511
BP_dia baseline–pyramid (*N* = 9)	1, 7	0.333	0.5818	1, 7	0.000	1.0000
BP_dia stereo–sphere (*N* = 19)	1, 17	1.638	0.2177	1, 17	1.564	0.2280
BP_dia stereo–pyramid (*N* = 9)	1, 7	0.000	1.000	1, 7	0.027	0.8752
BP_dia sphere–pyramid (*N* = 9)	1, 7	1.296	0.2924	1, 7	0.605	0.4622

Significance level ^*∗*^0.01 < *p* ≤ 0.05; ^*∗∗*^0.001 < *p* ≤ 0.01; ^*∗∗∗*^*p* ≤ 0.001.

## Data Availability

The data used to support the findings of this study are included within the article. Additional data are available from the corresponding author upon request.

## Abbreviations

2D: Two-dimensional
3D: Three-dimensional
ABI: Acquired brain injury
ADHD: Attention deficit hyperactivity disorder
ANOVA: Analysis of variance
ANS: Autonomic nervous system
BCI: Brain-computer interface
BDI: Beck depression inventory
BPM: Beats per minute
DPSS: Discrete prolate spheroidal sequences
EEG: Electroencephalogram
fMRI: Functional magnetic imaging
GLM: General linear model
GS: Geometric sound
HR: Heart rate
HRV: Heart rate variability
ICA: Independent component analysis
MDD: Major depressive disorder
MDMQ: Multidimensional mood questionnaire
NBS: Network-based statistic
SPL: Sound pressure level
TBI: Traumatic brain injury
TSITS: The Sound Is the Scenery
VCM: Voice coil motor
VR: Virtual reality.

## Supplementary Materials

Supplementary Materials S1 Supplementary 1: Sound Samples & Data: https://osf.io/y3ef2. S2 Supplementary 2: Custom Questionnaire: https://osf.io/nmjts. S3 Supplementary 3: Connectivity Patterns at Sporadic Frequencies: https://osf.io/awrsq. S4 Supplementary 4: General Free Testimonials EX1 + EX2: https://osf.io/qbz3g.
