# Supplementary material for: Effects of Geometric Sound on Brainwave Activity Patterns, Autonomic Nervous System Markers, Emotional Response, and Faraday Wave Pattern Morphology
Source: Evid Based Complement Alternat Med. 2024 Mar 29;2024:9844809. doi: 10.1155/2024/9844809 (PMC10997421; doi:10.1155/2024/9844809)
Supplement: Supplementary Materials — S1 Supplementary 1: Sound Samples & Data: https://osf.io/y3ef2. S2 Supplementary 2: Custom Questionnaire: https://osf.io/nmjts. S3 Supplementary 3: Connectivity Patterns at Sporadic Frequencies: https://osf.io/awrsq. S4 Supplementary 4: General Free Testimonials EX1 + EX2: https://osf.io/qbz3g. [file 9844809.f1.zip › Supplementary 1_Sound Samples & Data.....docx]

**Effects of Geometric Sound on Brainwave Activity Patterns, Autonomic Nervous System Markers, Emotional Response and Faraday Wave Pattern Morphology

Supplementary 1 - Sound Samples & Data**

Audio File 1 – Stereo (control): <https://osf.io/92yze>

Audio File 2 – Pyramid GS: <https://osf.io/pjc5k>

Audio File 3 – Cube GS: <https://osf.io/hn3bm>

Audio File 4 – Sphere GS: <https://osf.io/ubfwg>

Max dBA Levels: <https://osf.io/ex4yf>
