# Supplementary material for: Effects of Geometric Sound on Brainwave Activity Patterns, Autonomic Nervous System Markers, Emotional Response, and Faraday Wave Pattern Morphology
Source: Evid Based Complement Alternat Med. 2024 Mar 29;2024:9844809. doi: 10.1155/2024/9844809 (PMC10997421; doi:10.1155/2024/9844809)
Supplement: Supplementary Materials — S1 Supplementary 1: Sound Samples & Data: https://osf.io/y3ef2. S2 Supplementary 2: Custom Questionnaire: https://osf.io/nmjts. S3 Supplementary 3: Connectivity Patterns at Sporadic Frequencies: https://osf.io/awrsq. S4 Supplementary 4: General Free Testimonials EX1 + EX2: https://osf.io/qbz3g. [file 9844809.f1.zip › Supplementary 2_Custom Questionnaire....docx]

**Effects of Geometric Sound on Brainwave Activity Patterns, Autonomic Nervous System Markers, Emotional Response and Faraday Wave Pattern Morphology

Supplementary 2 - Custom Questionnaire**
**Sound Experiment Survey
Pre Session**

1. **How would you rate your happiness level this week?**

(1 being not good, 3 being neutral, 5 being excellent)

1 2 3 4 5

1. **How would you rate your confidence level this week?**

1 2 3 4 5

1. **How would you rate your depression level this week?**

1 2 3 4 5

1. **How would you rate your fear level this week?**

1 2 3 4 5

1. **How would you rate your relaxation level this week?**

1 2 3 4 5

1. **How would you rate your frustration level this week?**

1 2 3 4 5

1. **How would you rate your hopefulness level this week?**

1 2 3 4 5

1. **How would you rate your energy level this week?**

1 2 3 4 5

1. **How would you rate your physical condition this week?**

1 2 3 4 5

1. **Is there specific discomfort in your life that you would like to improve?** If so please explain

# Post Session

1. **How would you rate your happiness level after the session?**

(1 being not good, 5 being excellent)

1 2 3 4 5

- - Did you notice any change following the session? If so please explain:

1. **How would you rate your confidence level after the session?**

1 2 3 4 5

- - Did you notice any change following the session? If so please explain:

1. **How would you rate your depression level after the session?**

1 2 3 4 5

- - Did you notice any change following the session? If so please explain:

1. **How would you rate your fear level after the session?**

1 2 3 4 5

- - Did you notice any change following the session? If so please explain:

1. **How would you rate your relaxation level after the session?**

1 2 3 4 5

- - Did you notice any change following the session? If so please explain:

1. **How would you rate your frustration level after the session?**

1 2 3 4 5

- - Did you notice any change following the session? If so please explain:

1. **How would you rate your hopefulness level after the session?**

1 2 3 4 5

- - Did you notice any change following the session? If so please explain:

1. **How would you rate your energy level after the session?**

1 2 3 4 5

- - Did you notice any change following the session? If so please explain:

1. **How would you rate your physical condition after the session?**

1 2 3 4 5

- - Did you notice any change following the session? If so please explain:

1. **How would you rate your belief in sound healing?**

(1 being not at all, 10 being very much)

1 2 3 4 5 6 7 8 9 10

1. **Please describe in your own words your experience during the session (thoughts, images, emotions, areas in the body you could feel, colores etc’ if you had any)**
