# Supplementary material for: Effects of Geometric Sound on Brainwave Activity Patterns, Autonomic Nervous System Markers, Emotional Response, and Faraday Wave Pattern Morphology
Source: Evid Based Complement Alternat Med. 2024 Mar 29;2024:9844809. doi: 10.1155/2024/9844809 (PMC10997421; doi:10.1155/2024/9844809)
Supplement: Supplementary Materials — S1 Supplementary 1: Sound Samples & Data: https://osf.io/y3ef2. S2 Supplementary 2: Custom Questionnaire: https://osf.io/nmjts. S3 Supplementary 3: Connectivity Patterns at Sporadic Frequencies: https://osf.io/awrsq. S4 Supplementary 4: General Free Testimonials EX1 + EX2: https://osf.io/qbz3g. [file 9844809.f1.zip › Supplementary 3_Additional Connectivity Patterns.....docx]

Effects of Geometric Sound on Brainwave Activity Patterns, Autonomic Nervous System Markers, Emotional Response and Faraday Wave Pattern Morphology

Supplementary 3

Connectivity Patterns Observed at Sporadic Frequencies

Significant connectivity patterns were also observed at more sporadic frequencies: Stereo and Cube at 22Hz and Pyramid at 24Hz (Image 1A); Pyramid at 56Hz and 58Hz. These show close proximity in frequency range and on similar or different GS conditions and might be indeed significant. The connectivity pattern at 58Hz specifically is showing a symmetric pattern in Pyramid GS between the left and right hemispheres, creating a triangular pattern (Image 1B). Other frequencies showing significant connectivity patterns compared to base condition were Pyramid GS at 48Hz, 66Hz, 74Hz. It is interesting to note that as the frequency of brain waves go up most connectivity patterns activity is measured on the right hemisphere (Image 1C-1C).


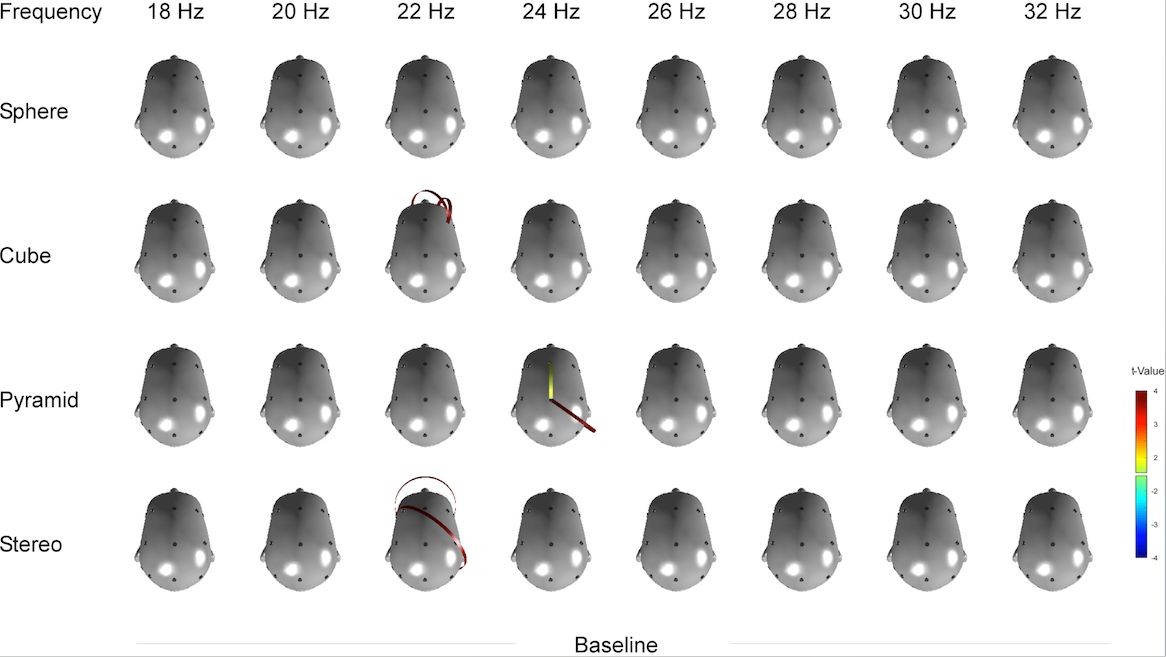


**Image 1A:** Statistically significant connectivity patterns compared to base condition. Stereo and Cube at 22Hz and Pyramid at 24Hz


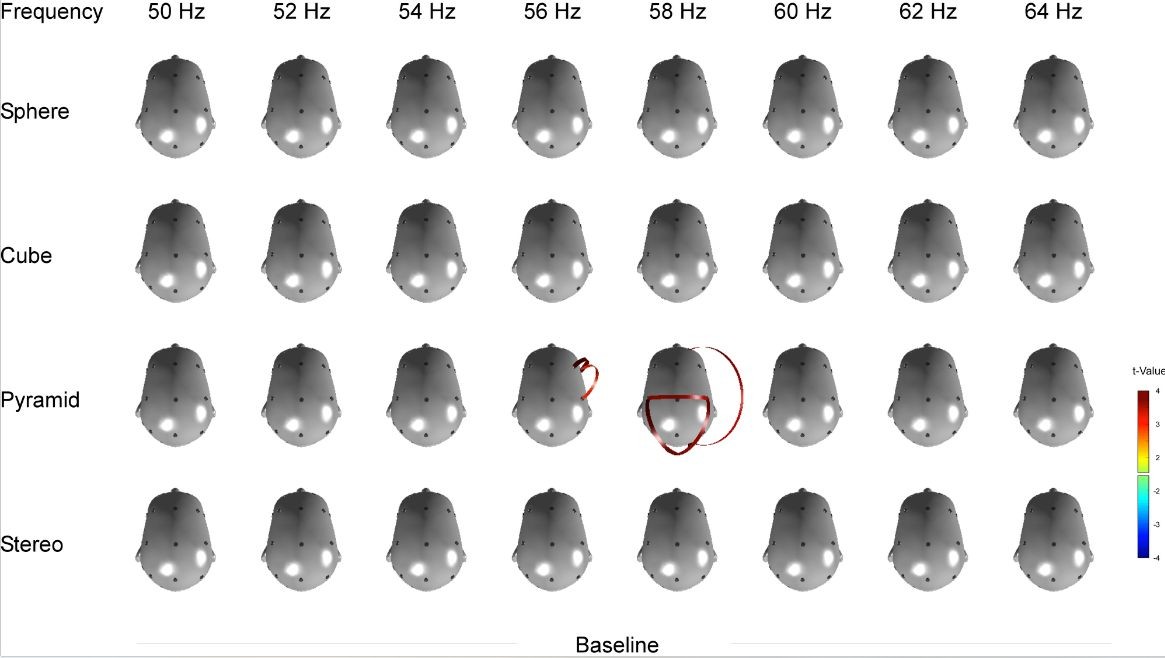


**Image 1B:** Statistically significant connectivity patterns compared to base condition. Pyramide at 56Hz and 58Hz


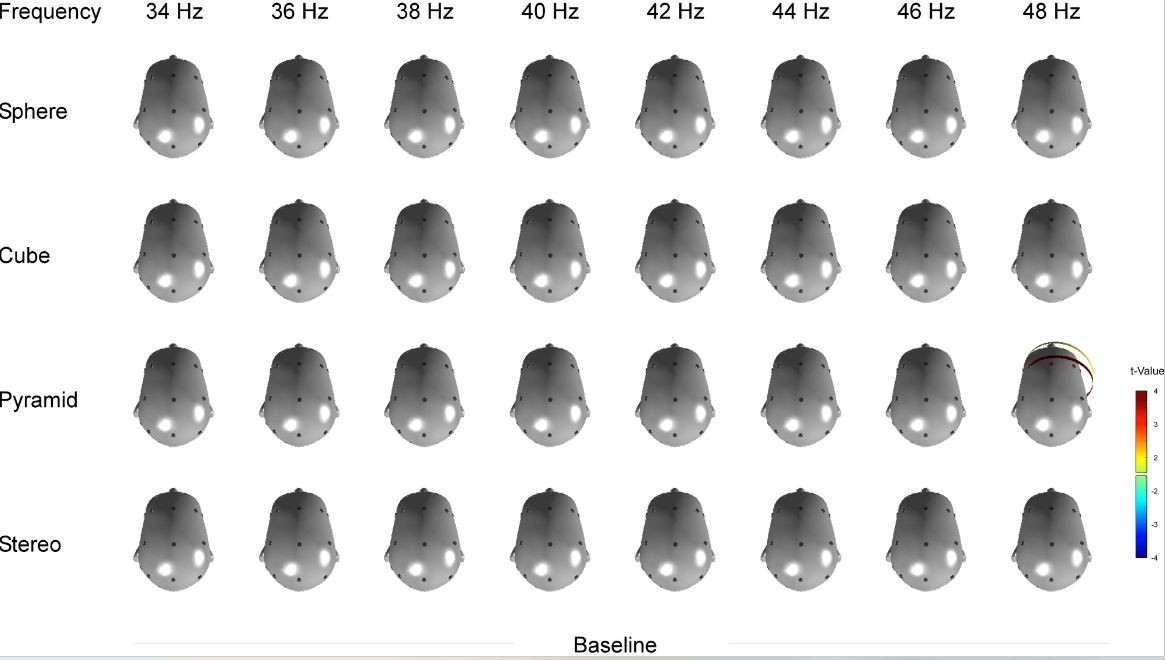


**Image 1C:** Statistically significant connectivity patterns compared to base condition. Pyramide at 48Hz.

Statistically significant connectivity patterns compared to base condition in sephoradic frequencies (Pyramid at 66Hz, 74Hz, 124Hz ; Cube at 82 Hz, 92Hz, 126Hz ; Stereo at 108Hz). These patterns were detected above threshold noise at 64 Hz. A closer examination using different filters is to be employed in the future to confirm activity over 64Hz. Images are brought here for observation and were not used in conclusion for research results (Figures 2A-2D).


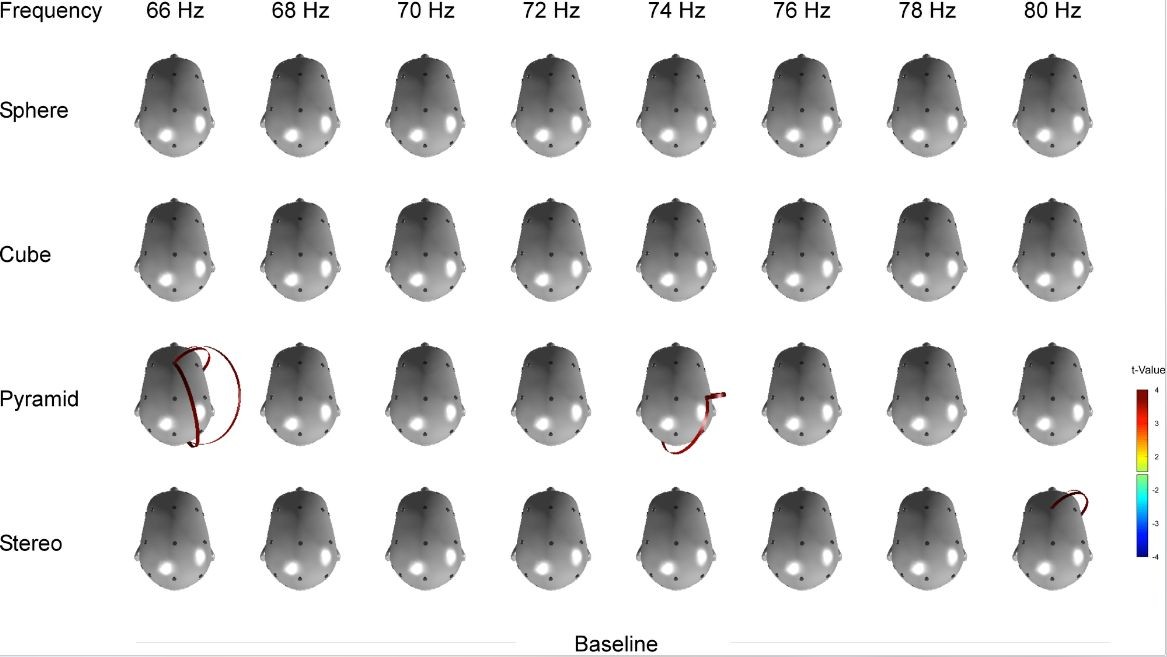


**Figure 2A:** Statistically significant connectivity patterns compared to base condition. Pyramide at 66Hz AND 74Hz.


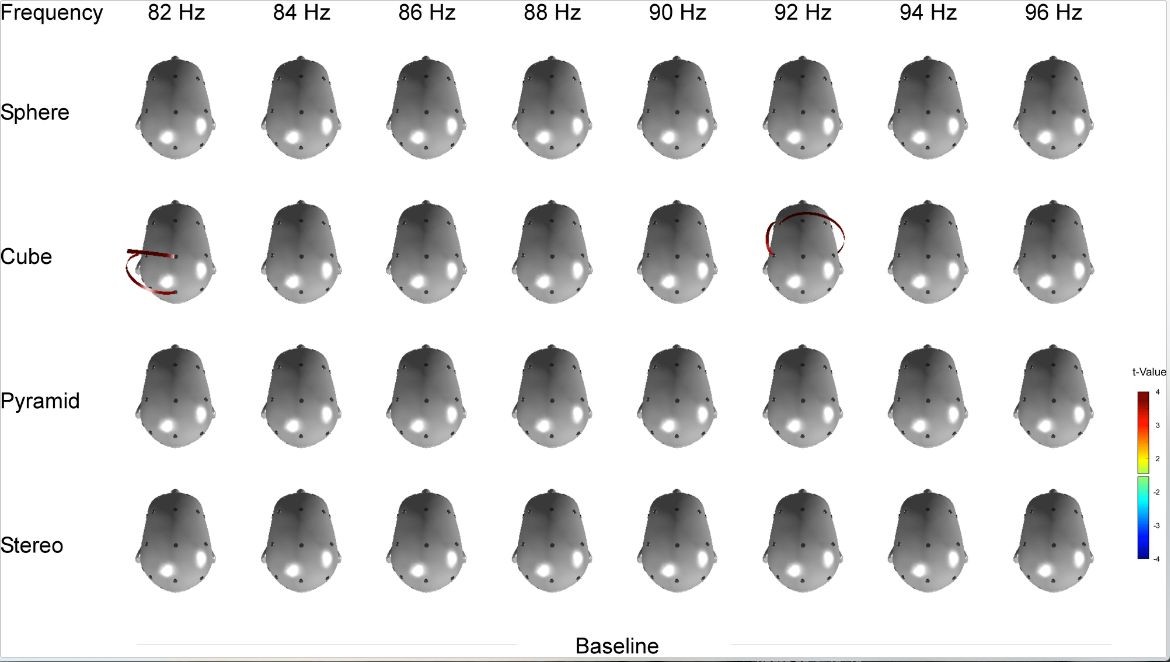


**Figure 2B:** Statistically significant connectivity patterns compared to base condition. Cube at 82Hz and 92Hz.


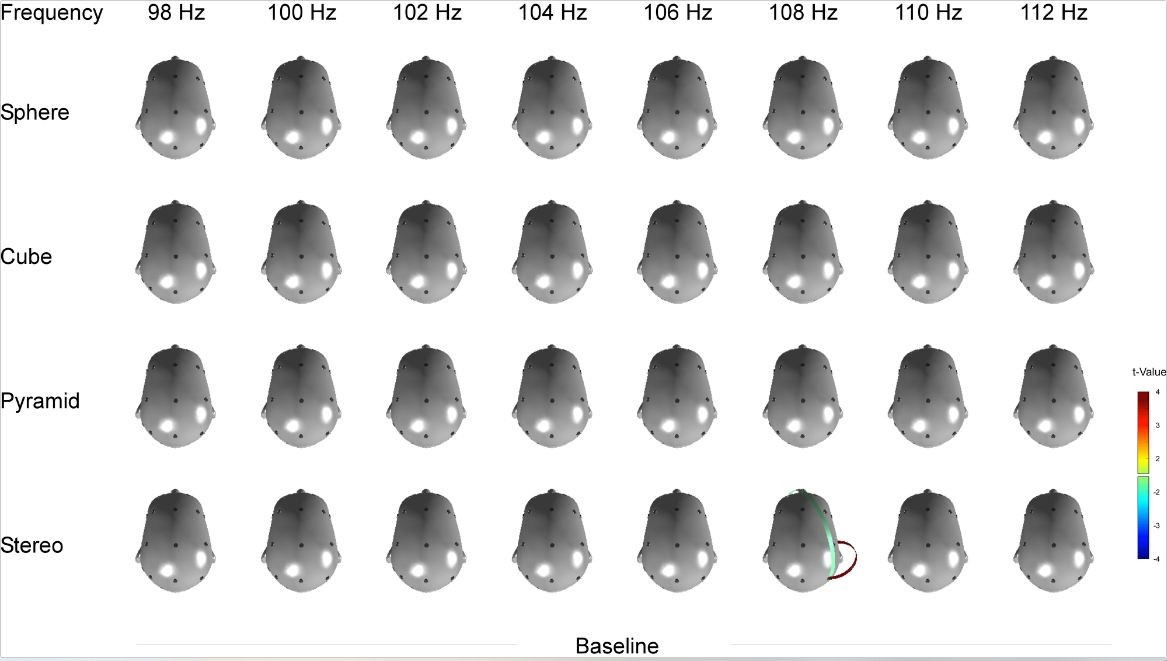


**Figure 2C:** Statistically significant connectivity patterns compared to base condition. Stereo at 108Hz.


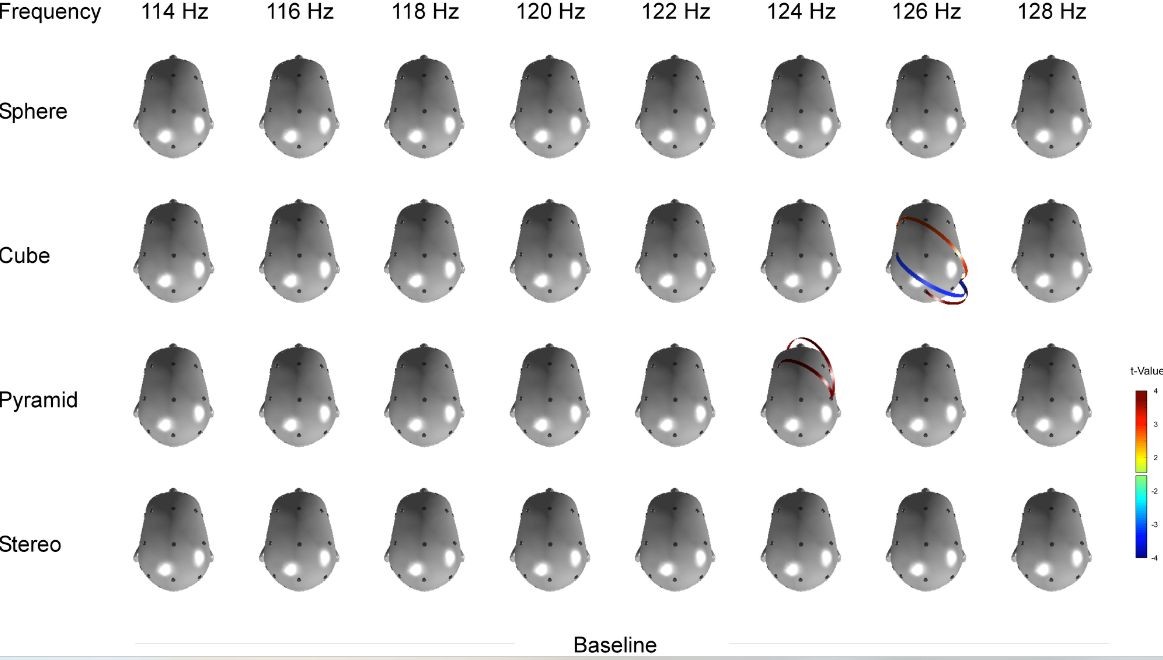


**Figure 2D:** Statistically significant connectivity patterns compared to base condition. Pyramide at 124Hz and Cube at 126Hz.
