# Supplementary material for: Effects of Geometric Sound on Brainwave Activity Patterns, Autonomic Nervous System Markers, Emotional Response, and Faraday Wave Pattern Morphology
Source: Evid Based Complement Alternat Med. 2024 Mar 29;2024:9844809. doi: 10.1155/2024/9844809 (PMC10997421; doi:10.1155/2024/9844809)
Supplement: Supplementary Materials — S1 Supplementary 1: Sound Samples & Data: https://osf.io/y3ef2. S2 Supplementary 2: Custom Questionnaire: https://osf.io/nmjts. S3 Supplementary 3: Connectivity Patterns at Sporadic Frequencies: https://osf.io/awrsq. S4 Supplementary 4: General Free Testimonials EX1 + EX2: https://osf.io/qbz3g. [file 9844809.f1.zip › Supplementary 4_General Free Testimonials .....docx]

**Effects of Geometric Sound on Brainwave Activity Patterns, Autonomic Nervous System Markers, Emotional Response and Faraday Wave Pattern Morphology

Supplementary 4 - General Free Testimonials EX1+EX2**

**E1 - Post Session:**

# (Replies numbers are in conjunction with items in Custom Questionnaire provided in Supplementary 2)

●

5. Feeling more relaxed / sleepy

8. A bit more tired

11. I felt kind of free at a center point even “thoughtless”

●

1. Its not that I am less happy but there was something that settled within me that opened me up some up that was a bit of sadness.

5. Yes, it was a “soothing” experience mostly to the sound.

8. Since I am more relaxed I feel like I could take a nap.

11. I mentioned it briefly at the beginning of the questionnaire but I felt an expansiveness from the gongs. My mind began to expand and that allowed thoughts to creep in. Right now I'm very busy with work so a lot of items come to mind, like a list, but when the gong would sound, I was able to pull back into focus.

●

1. Calmer

4. During the experiment I was a little nervous but was ok in the end.

5. Not moving; calming sounds and isolation

11. Images of soundwaves; association to meditation

●

5. Could have even fallen asleep.

1. Feeling less energetic.

9. Feeling tired but headache went away.

11. Was thinking if I should concentrate on the sound or not following the sound “running” around my body red circles moving around and back together in front of my eyes.

●

1. I was getting more and more relaxed, close to falling asleep.

3. Being relaxed made me worry less.

5. (same as question 1)

1. Im looking more optimistic to my day.
2. Being relaxed gave me more motivation to do what I was up to today.
3. I am really content.

11. I was seeing people I don't know and had conversations with them or followed their conversations. I cant remember what they were about.

Sometimes there were special people. E.g. with a painted face but it seemed normal. I also went through conversations I am gonna have today and it made me feel secure.

At other points I felt I was almost falling asleep and thought “was I supposed to listen to the sound actively?” But I couldn't help it, it happened again and again. It almost felt like being in a deep meditation.

●

5. The session helped me forget for a moment about task that I was worried from the morning. So, and after the session I can think about the tasks from scratch.

11. I was thinking about relaxed (relaxing) places that I visited before, and where I had a quiet moment to switch off logical thinking and merge with the environment.

●

1. First it felt like the sound was coming from one place, the other times I noticed different sounds from different locations. But I couldnt tell if the third time was different from the second.
2. Not really, always great to follow sound.

7. Sounds were quite soothing

11. I felt calm.

I saw colors: purple and yellow moving into each other.**

Wasnt noticing any difference in the body though but probably because it was a bit cold

** Only in the 2nd and 3rd time the purple and yellow were like spots.

●

2. More relaxed and focused.

●

1. During the second session I felt a bit irritated.

9. My neck feels quite tight.

11. The first time I felt a bit apprehensive by the second a tad irritated but by the third I felt very relaxed.

●

5. I feel that I have more focused energy.

11. It was like meditating, like a sound massage. Many images and thoughts passed through my head but in the end it does not matter anymore.

●

Very relaxed and happy.

1. Much more relaxed than before the session.

11. Feeling of deep relaxation.

●

There was a moment to process the present - really consider the state of things.

1. Better after processing (which always helps me).
2. Definitely calmer.
3. Totally
4. Noticeable less agitated by everything.
5. Also noticeable more realistic about everything.
6. A bit tired.
7. Need some serious body work - sensed that in the session.
8. As long as you’re listening actively
9. Felt all areas of my body that were tensed or misaligned and got a bit emotional in the middle of the session thinking of some personal situations.

●

11. Light / visual snow that formed the movement of the tone.

My imagination wandered for a minute because I got a little bored of the repetitive sine tone. I started imagining other music in my head...

The sound evolved a singing bowl but without subtle timbre and tone variation of a real singing bowl in real time.

I felt somewhat of a phantom “hand” wavering around my head in the place where the sound travelled.

Color was more white/faint orange (visual snow / glow that splotches With location of the sound)

●

11. Lots of memories from the past days.

●

1. More relaxed.

Smiling unconsciously, eager

1. More enthusiastic I guess.
2. I feel calm and patient now.
3. I trust the rest of the day will go well.
4. Energetic but also very very relaxed (drowsy)

11. Also twice very precise itches in the right foot and left finger. Ring of sound going around my head. Very pleasant in the back of the head, calming. Eyes almost revolting Like in meditation.

●

1. I feel more vitality and confidence.
2. I think less about circemstansous and I feel more spontaneous.
3. I was a little worried during the experiment but now that feeling disappeared.
4. I am relaxed but also active.
5. I feel “sparkling”.
6. I feel more relieved, also physically and possibly more elastic.

●

1. Yes, possibly due to a relaxed state of mind after breathing calmly.
2. Yes, afterwords I felt replenished.
3. -“-
4. -”-
5. -”-

●

2. Sound & music generally relaxes me and my overall feeling / my spirit get lifted.

●

1. Not really, it was quite relaxing.

5. It was slightly like meditation.

11. I was focusing on the source of the sound. Felt like Im following it with my eyes. It was relaxing, reminded me of the meditation of yoga class.

●

1. Generally somewhat improved due to how the experience affected the other factors.

5. The nature of the sound that was played helped endure a state of relaxation; meditative.

11. I have experience in meditation and it helped me to get quicker into a state of higher concentration. I could feel tingling sensations throughout the body , but that cant be attributed to the sonic experience entirely.

●

# E2 - Post Session:

**(Replies numbers are in conjunction with items in Custom Questionnaire provided in Supplementary 2)**

1. I feel happier having experienced the experiment. It was interesting and cool
2. I feel more confident and overall more relaxed with the environment.

5. The doctors voice was relaxing, made me feel more at ease.

1. Meditative, solitude, introspective, metallic/silver, metal prong being sruk.

●

1. I feel happy and relaxed, much calmer after than before.
2. It was very relaxing so maybe that makes you feel more confident.
3. I dont feel depressed - I did not before either.

5. Very Relaxed.

1. A bit sleepy due to sitting relaxing.
2. I feel good, like I could enjoy a walk in the sun.
3. I think it depends on the state you are going in. If you are in full panic mode I dont think it helps but if you start from a calm place maybe yes.
4. I felt like I was almost falling asleep intensely like a sudden “falling” feeling in my head. My thoughts were almost entirely focused on the location of the sound - though drifting at a few moments too.

●

11. My brain was somewhere else, I felt completely disconnected.

●

1. I feel a bit better because the session made me look inside me, take a break from a hectic week and reset myself.
2. I feel a bit more confident as I had time to strengthen my inner me during the session.
3. I didnt have any negative thoughts during the session, before I had some in mind.
4. Before the session I had some nervousness/fear of the unknown (what will happen inside?) and during the session I lost that fear.
5. It calmed me down and made me sleepy.
6. I feel more positive in general.
7. I feel more sleepy and not physically active.
8. I feel more tired.

11. At the beginning my mind was full of images and thoughts about unrelated things. In the first parts the sound helped me focus on myself and try to forget all those other thoughts.The sound “dongs'' created a small explosion in my head, right side that spread all over my body. From head down to shoulders and arms and body and fingers. I was seeing white sometimes. I feel

asleep in the last part. I got very sleepy and started “dreaming” - I forgot the dreams the moment I “woke up” every time. When I was awake I tried to not “daydream” and just focus on myself. I started feeling just good and comfortable, I didn't feel my body anymore.

●

1. The self reflexiveness I found at first diminished.
2. I feel at ease and quite peaceful.
3. Slight but I'm feeling positive.

9. I am relieved and recharged.

11. A blue sphere outlined in black.

●

1. Emotions are more fluent than before.
2. It became better because the inner persevere has gone.

5. There’s a feeling of being more connected to the world.

11. Abstract, fluent, ambient color waves/shifts, positive emotions, feeling of being outside the body, but a heavy head sometimes. Colors: mostly green, purple, blue, orange.

●

4. Sounds helped me calm and see the good rather than the bad.

5. Monotonous sounds + closed eyes are relaxing.

9. Body and mind are so relaxed, would need drive a car now.

●

1. Clearer headspace, less stress.
2. The session felt like a long good night’s sleep, I feel very euphoric and positive.
3. As stated above I feel fully recharged after the session.
4. No Fear.
5. I feel as if all the stress has been elevated (removed).
6. I feel less frustrated than normal.
7. I can not exactly state what is different , but positive outcomes and mind sets are higher than before the sesion.
8. I feel energetic, alive and aware, less restless.
9. The session was very relaxing and powerful. Many feelings of positive arose during and after the session. During the 2nd and 3rd sound samples the most effective results were achieved.

●

1. I was feeling pretty great already but the relaxation makes it more obvious.

5. Definitely more relaxed and calm body and mind.

1. Perhaps everything seems a bit more hopeful now.
2. A bit higher (*energy level).
3. Calmer and more relaxed.

11. It was very similar to meditating, with a focus on the sound. My body relaxed more and more to the point I would not feel my arms or legs as I kept still. Mind would clear from thoughts on occasion.

●

5. I feel a bit tired and unconcentrated.

11. After the session I feel a bit dizzy, uncomfortable. Thoughts:

I didn't have any specific thoughts, they stayed mostly in the room and about the experiment and my daily life.

Images:

I didn't have images, maybe some waves in front of my closed eyes. But I rarely to never see pictures from imaginations.

Emotions:

I didn't feel a shift in the experiment. Body:

Was comfortable and resting, with some slight itching at nose and face at some point.

●

1. Less worry for everything.

1. Yes, Im like narcotized, like when you take medication for allergy (anti-staunics).
2. Yes, anything matters now.
3. Yes, everything looks easier.

9. I feel tired now.

11. I had hallucinations/dream made by ankles, going big and then going little, like pulsating circles. Like a watermark. I fell asleep.

●

1. Sound went from stereo to more channels - sound moved through the room. Happiness level was not changing *(Participant rated happiness level at 4 pre experiment and 5 post experiment. RG)*

1. Curiosity started so the depression level went down but didn't feel depressed before.
2. Level changed from a little fear of the unknown to no fear when the experiment started.
3. Relaxation went up when the experiment started.
4. Even though I didn't know what to expect I had something different in mind - Frustration level didn't change much.
5. Went up when more channels started playing.
6. Energy level went a little down from closed eyes - went up when more sound channels started.
7. Feeling more relaxed.

11. Felt somehow like being in the nature since the sound was moving.

●

11. It was very interesting & exciting. My body reacted to the movement of the sounds (especially arms and legs).

●

11. I kind of visualised the sound as a line, light line cross my view from right to left. I had some thoughts but it was easy to focus back on the sound. My neck, head and shoulders were the parts of my body I could feel aware. At some point I forgot the exact position of my hands. I really wanted to look at them when I finished. The thoughts I had were about my professional future. It wasn't about worrying, more like resolutive thoughts.

●

1. Still quite happy :)
2. I am not so confident about was has been achieved but looking forward to see the next steps.
3. I am still quite concentrated about other thoughts after spending some time thinking of them.

5. I enjoyed the time of letting go and following the sound.

7. Quite high *(hopefulness. RG)* about the future.

9. A bit unaware it was more mental for me.

●

5. Much more relaxed.

11. Tingling sensations in arms and hands. No olores.

●

11. I am having more thoughts

●

1. More aware of what Im feeling/thinking.

1. Feeling a little calmer after the session.
2. Slightly less fear, perhaps also due to the experience …… with people
3. I do feel a little more relaxed after the session. It was very pleasant conversations and exchange with the people there. Also the modern life we live seldom allows the chance of sitting somewhere concentrated for a or just a concentrated listening session.
4. Slightly less frustrated, the worries are still there but I guess this helped me to realize that problems can be put aside for a little to enjoy what is going on.

8. Not much changed for energy level because I was tired to begin with but I was more concentrated.

11. Partly of what I answered in question 5 can go into here. I feel more aware of my hands and (deep relaxation on hands, strangely) and my head.

●

1. I felt more relaxed.

1. I feel more relaxed as a result of the last round of circular music, which calmed me considerably.
2. I am more relaxed and at ease than before.
3. I am less frustrated as a result of the calming atmosphere and faux-meditation.

8. I feel more energetic after a period of restful focus.

11. Each beat was the shape of a glowing orb dropping on the left. At the end the orb rolled in a square around me, from left to back to right to front. The only physical sensation was a jolt of awareness when a sudden beat after a period of silence occured. I found the music to be relaxing overall, with moments of slight tension.

●

5. Relaxed - almost fell asleep (often meditate so its no surprise).

8. Almost fell asleep - lower energy level.

11. Certain sounds triggered certain areas in the brain. I was generally very calm. I could hear other talking but I'm detached. I was seeing visions mostly I couldn't remember. One thing I remember was I was looking behind a guy’s shoulder and I saw he’s operating stuff on the computer. I thought, is this Frank?

●

11. The headcup was uncomfortable for my clim. I wanted to pee a for and didn't feel comfortable because of that.

●

11. I felt a deep meditation state.

●

1. Yes, I feel happier.

1. Yes, more relaxed.

11. Enjoyable trip through healing paradise at ca. 440hz

●

1. I felt gradually more relaxed.

3. I got to kind of deep meditation and helped me out with my overall tension.

1. I felt slightly more motivated.
2. As a consequence from the previous answer.

11. Lots of thoughts coming and going life waves and moments of pure relaxation / visualization of sound as waves.

●

1. I feel more calm, meditative.

11. Relaxing, I could visualize many wavy shapes all different for each sample. It was also very meditative.

●

3. Deep calm down.

11. Felt more in a gong session at iboga Less till nothing emotions.

●

11. Relaxation.

I had the feeling the sound is going through my body.

●

1. A little happier after the session.
2. A bit more confident after.

11. Close to a meditation experience where I was going through a lot of thoughts with no images and relaxed up until the 3rd track where I went into a shock mode, I started seeing visuals and my body became more tense, I was resisting opening my eyes
